# An interaction-motif-based scoring function for protein-ligand docking

**DOI:** 10.1186/1471-2105-11-298

**Published:** 2010-06-02

**Authors:** Zhong-Ru Xie, Ming-Jing Hwang

**Affiliations:** 1 Institute of Biomedical Informatics, National Yang-Ming University, Taipei 112, Taiwan; 2 Institute of Biomedical Sciences, Academia Sinica, Taipei 115, Taiwan

## Abstract

**Background:**

A good scoring function is essential for molecular docking computations. In conventional scoring functions, energy terms modeling pairwise interactions are cumulatively summed, and the best docking solution is selected. Here, we propose to transform protein-ligand interactions into three-dimensional geometric networks, from which recurring network substructures, or network motifs, are selected and used to provide probability-ranked interaction templates with which to score docking solutions.

**Results:**

A novel scoring function for protein-ligand docking, *MotifScore*, was developed. It is non-energy-based, and docking is, instead, scored by counting the occurrences of motifs of protein-ligand interaction networks constructed using structures of protein-ligand complexes. *MotifScore *has been tested on a benchmark set established by others to assess its ability to identify near-native complex conformations among a set of decoys. In this benchmark test, 84% of the highest-scored docking conformations had root-mean-square deviations (rmsds) below 2.0 Å from the native conformation, which is comparable with the best of several energy-based docking scoring functions. Many of the top motifs, which comprise a multitude of chemical groups that interact simultaneously and make a highly significant contribution to *MotifScore*, capture recurrent interacting patterns beyond pairwise interactions.

**Conclusions:**

While providing quite good docking scores, *MotifScore *is quite different from conventional energy-based functions. *MotifScore *thus represents a new, network-based approach for exploring problems associated with molecular docking.

## Background

Drug design and discovery play a pivotal role in driving research in computational chemistry and biology [1-6]. In computational drug design and discovery, it is often necessary to determine, as a first step, the binding of a ligand to a target protein. The computational scheme for predicting ligand binding occurrence, affinity, and orientation is commonly referred to as "molecular docking", which has been a topic of intensive research for decades [6,7]. The development of a molecular docking tool usually starts with an efficient search algorithm, which places the ligand in the active site of the target protein in numerous different positions, orientations, and, in flexible docking, conformations. These are then evaluated by a scoring function to distinguish between good (near-native) and bad (decoy) docking solutions. The two aspects of searching and scoring can be, and usually have been, developed and evaluated separately, although one clearly affects the other and a balance is often sought to meet specific study aims [6,8,9].

Scoring functions for molecular docking are traditionally either physics-based or knowledge-based [6,9] and differ mainly in the derivation of the mathematical models used to compute the energies of molecular interactions. Physics-based scoring functions employ a set of molecular interaction terms to compute binding energies. For example, the scoring function used in G-Score [10,11] or AutoDock 3.0 [12] is based on the molecular mechanics force field used in Tripos [13] or Amber [14], respectively, and F-Score [15] and ChemScore [16] derive the coefficients of their energy terms using regression analysis of experimentally determined binding energies [17]. In contrast, knowledge-based scoring functions, such as PMF [18] and DrugScore [19,20], rely on statistical observations of preferred protein-ligand contacts, from which binding energies are calculated.

A common thread in these scoring functions is that, with few exceptions, they all operate on the assumption that the total interaction will be faithfully represented by an additive summation over a series of pairwise interactions between interaction centers, which are either real (such as individual atoms) or imaginary (such as the geometric center of a group of atoms). Despite fundamental fallacies in this assumption, it has been adopted for many years, as otherwise the problem would be intractable or too computationally expensive to handle practically [21,22].

In this study, we investigated a new idea for developing a scoring function for molecular docking. The novelty of the idea lies in the use of a network approach to identify frequently occurring patterns of protein-ligand interactions not in the form of pairwise interactions, but in the form of network motifs, where "network" refers to a collection of pairwise interactions. The motifs, which are assemblies of multiple pairwise interactions between proteins and ligands that are frequently observed in a database of known protein-ligand complex structures, thus represent energetically favorable ways of positioning a ligand molecule in the active site of a protein. Such a motif-based approach offers a new line of exploration for research on molecular docking. As a first step in this line of research, we present the construction of a motif-based scoring function, called *MotifScore*, and the evaluation of its ability to distinguish between good and bad docking solutions. Our results using a benchmark dataset showed that *MotifScore *performed well against a number of scoring functions used in existing docking programs.

## Methods

In constructing the protein-ligand interaction network, the three-dimensional (3D) coordinates of a set of protein-ligand complexes were each transformed into an atom-atom interaction network. There are two types of nodes in the network, namely atoms (interacting centers) from the protein and the ligand. Two nodes (one from the protein and the other from the ligand) are connected by an edge if their interaction is deemed significant by a distance threshold determined from a statistical analysis of a dataset of protein-ligand complexes (see below). Interactions between atoms within the protein or within the ligand are not considered. These networks were then broken down into many interaction motifs representing simple units of the network with specific protein-ligand interaction patterns.

### Datasets

To establish distance thresholds for constructing the protein-ligand interaction network, we performed a large-scale statistical analysis on a diverse set of known structures of protein-ligand complexes. The available protein-ligand complex structures as of January 10, 2006 in the Protein Data Bank (PDB) [23] were screened. To simplify our task, only those determined by X-ray crystallography and with only one ligand molecule were selected. Furthermore, those containing DNA/RNA molecules and those in which the ligand binds to its target protein covalently were discarded. Complexes containing a heme group were also excluded because one may consider the heme group as a part of the protein, rather than a ligand. Solvent molecules and ions, such as chloride or ammonium, that are often included to facilitate crystallographic studies were not considered as ligands, whereas metal ions such as zinc or calcium ion that are coordinated by metal-binding amino acids were considered as an extension of the protein molecule, and their interactions with ligand were considered part of the protein-ligand interaction network constructed. In all, we obtained a total of 6,276 structures of complexes that could be used in the statistical analysis.

This set of PDB protein-ligand complex structures is not entirely non-redundant since a single protein or its mutants may be co-crystallized with many similar compounds, and, conversely, the same compound may be co-crystallized with multiple homologous proteins. However, unless both the protein and the ligand are completely identical, part of the resulting interaction networks can still be distinct, and as defining non-redundant networks is not a straightforward task, we chose to include as many network connectivities (i.e. protein-ligand interactions) as possible while conducting normalizations to minimize the potential bias of using a redundant dataset. Furthermore, analysis on a reduced set (4,190 structures) by excluding those that share >25% protein sequence identity and identical ligand showed that the resulting network motifs and their grades (defined below) had a Pearson's correlation coefficient of 0.95 with those of the set of 6,276 structures (data not shown), suggesting that including homologous proteins and identical ligand compounds would not significantly affect the outcome of our analysis.

Besides the set of 6,276 structures, we used a subset of the LPDB (Ligand-Protein DataBase) [24], which contains decoys generated from molecular dynamics simulations [8], to optimize the parameters of our scoring function, *MotifScore*. There are more than 200 complexes, each with a set of various numbers of decoys, in the LPDB, but only 113 of these were used in this work because we found that the ligand names, atom names, and the ordering of atoms of many of the decoys in LPDB were not the same as in the original data files in the PDB, making it difficult for us to correctly assign their designated atom types (see below). For testing and evaluating *MotifScore*, the data set of Wang et al. [9] was used. The Wang dataset consists of a set of 100 protein-ligand complexes, each of which comes with 100 docked conformations (i.e. decoys) generated using AUTODOCK 3.0 [12]. Since Wang et al. have used this dataset as a benchmark to evaluate a number of docking scoring functions [9,20], we used it to compare the performance of *MotifScore *with that of a number of other scoring functions.

### Atom type assignment

Based on the work of others [18,25] and the chemical properties of simple molecules, we created an atom type classification scheme to describe protein-ligand interactions. We defined a total of 23 atom types (Table 1), of which 14 were for protein atoms and 20 for ligand atoms, with many of them shared by both. The atom types were identified by a 3-code name, or a 3-letter name for those that did not need to be further classified, as they were relatively rarely observed in our dataset of protein-ligand complexes (e.g., metals, phosphorus, and halogens). Some general rules for the 3-code names were as follows. The 1^st ^code was the name of the element (C, N, O, and S) and the 2^nd ^and 3^rd ^code indicated the surroundings and electrostatic properties of the atom. The 2^nd ^code could be 2, 3, R, or L, which, respectively, correspond to sp2 or sp3 hybridization or inclusion in an aromatic ring or an aliphatic chain. The 3^rd ^code could be P, N, A, D, B, E, or C, which, respectively, correspond to polar, non-polar, hydrogen bond acceptor, hydrogen bond donor, an atom that can be both a hydrogen bond donor and acceptor or either a hydrogen bond donor or acceptor, or a charged atom. The 'either (E)' code was associated with the atom type NRE, which was used primarily for the two nitrogen atoms on the imidazole ring of a histidine, as both of the nitrogens can be either protonated (a hydrogen bond donor) or deprotonated (a hydrogen bond acceptor). For simplicity, the nitrogen of tryptophan, which is much infrequently seen than histidine especially in the active site, is also assigned the atom type NRE.

**Table 1:** Atom types and descriptions

Atom type	Description	Example^b^	On protein	On ligand
C2N	C, SP2, normal/non-polar	-C=C-		x
C2P	C, SP2, polar	-C=O	x	x
C3N	C, SP3, normal/non-polar	-C-C-	x	x
C3P	C, SP3, polar	-C-OH	x	x
CRN	C, aromatic, normal/non-polar	a benzene carbon	x	x
CRP	C, aromatic, polar	a halogenated aromatic carbon	x	x
O2A	O, SP2, hydrogen bond acceptor	>C=O	x	x
O3A	O, SP3, hydrogen bond acceptor	C-O-C		x
O3B	O, SP3, both	-OH	x	x
OLC	O, aliphatic, charged	-COO^-^	x	
ORA	O, aromatic, hydrogen bond acceptor	oxygen in furan		x
NLA	N, aliphatic, hydrogen bond acceptor	-NR_2_	x	x
NLB	N, aliphatic, hydrogen bond donor	-NH_1or2_	x	x
NLC	N, aliphatic, charged	-NH_3_^+^		x
NRA	N, aromatic, hydrogen bond acceptor	a non-protonated aromatic nitrogen		x
NRD	N, aromatic, hydrogen bond donor	a protonated aromatic nitrogen	x	x
NRE	N, aromatic, either H.B. donor or acceptor	nitrogen (protonated or not) of imidazole	x	
S3N	S, SP2, normal/non-polar	-SH	x	x
SRA	S, aromatic, hydrogen bond acceptor	sulfur in an aromatic ring		x
SLC	S, aliphatic, charged	-SO_4_^-2^		x
PHO	Phosphorus			x
MET	Metal^a^		x	
HAL	Halogen	F, Cl, Br, I		x

^a ^Metal ions, such as Zn, were considered in this work as an extended interaction site of the protein molecule.

^b ^See Fig. S3 in Additional file 1 for a representative chemical structure of the atom type CRN, CRP, ORA, NRA, NRD, NRE, and SRA.

In our scheme, each individual atom is itself an interacting center. For example, the chemical group PO_3 _would have four interacting centers: namely, one PHO (phosphorus) and three OLC (charged oxygen). Others have used pseudoatoms or pseudocenters (e.g., [26]), such as the geometric center of a chemical group, which may have the advantage of the resulting scoring function being less sensitive to the exact interaction distances used. On the other hand, chemical group centers do not fully account for the complex interactions, including the different interacting orientations, for example, of the constituent atoms. The interaction network motifs derived here represent a hybrid of both, since interactions are computed on real atoms but the scoring function is a non-energy-based network motif count, which does not rely on a precise range of interaction distances (see below). The interaction networks could also be constructed using pseudoatoms instead, but whether this would yield better results remains to be investigated.

For an automated atom type assignment, for protein molecules, we relied on standard PDB files, which have conventional names and thus the implicit bonding information for all protein atoms, and, for ligand molecules, we relied on the het dictionary file on the PDB website [23], which contains the complete naming and bonding information of all the ligand molecules that appear in the PDB files.

### Determination of atom-atom interaction thresholds

Whether or not there is an interaction between two atoms is determined by the energy produced by the interaction, which is affected by many variables, but mostly the distance between the two atoms. To save computational time, most studies have used distance instead of energy as the criterion to determine whether two atoms interact. The threshold of distance for interaction could greatly affect the complexity and outcome of a study, but most knowledge-based docking studies seem to suggest that a cutoff in the range of 4-6 Å between heavy atoms can achieve optimal performance [25]. However, a single cutoff may not be sufficient for the present study, because: 1) whereas conventional methods use a distance cutoff to avoid the computation of the less significant portion of interaction energies, we used the distance cutoff merely to establish network edges, which, unlike conventional scoring functions, do not involve a distance-dependent energy computation; 2) in the absence of a distance-dependent energy function, clashes between two interacting atoms cannot be prevented. In order to appropriately define atom-atom interactions (network edges), we therefore introduced not only an upper distance threshold for any pair of interacting atoms, but also a lower one. As described below, the values of these thresholds were determined by examining the distance distributions of all protein-ligand atom pairs (a total of 14 protein atom types × 20 ligand atom types, i.e. 280) in the 6,276 protein-ligand complex structures selected from the PDB.

**Figure 1:**
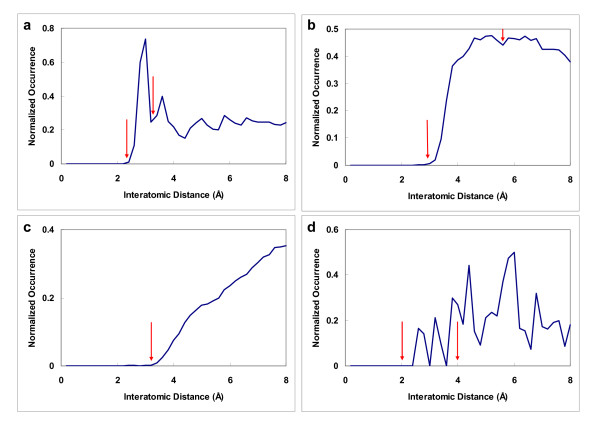
**Examples of the four categories of normalized distributions of protein-ligand interatomic distances**. The four represent strong interactions, weak interactions, non-specific interactions, and rare interactions (see text). Each is represented by a specific example, namely: **a) **a hydrogen bonding contact in the interaction of NRD with OLC; **b) **a van der Waals contact in the interaction of C2N with CRN; **c) **a non-specific contact in the interaction of C3N with C2P; and **d) **a rarely seen contact in the interaction of NLC with S3N. The red arrows mark the upper and lower distance cutoffs used to connect atoms in constructing the protein-ligand interaction networks: those separated by a distance within the upper and the lower threshold would be connected, while no connection was made for the non-specific interactions in the third category.

Following the concept of radial distribution [27], we analyzed the occurrence distribution of all atom type pairs as a function of separation distance. For atom type x, in order to determine the distance interval at which the highest density of atom type pair x-y occurs (hence the preferred interacting distance interval between x and y), we divided the occurrence of y by the difference between the cubes of the outer and inner radial distances from atom x (for example, 3^3 ^- 2^3^, when y is found between 2 and 3 Å from x, which is proportional to the volume of the shell within which y atoms are found). For normalization, we also divided the occurrence of y atoms found in this particular distance interval by the total occurrence of y atoms in the 6,276 complex structures in the PDB. The resulting distributions were quite distinctive from one atom type pair to another. However, to a large extent, they could all be characterized, albeit approximately, as one of four categories, as summarized in Fig. 1.

The first category (Fig. 1a) featured a sharp peak at a small distance, indicating a specific distance or a narrow range of distances for contact (i.e. interaction) between the two atom types. More than one-third of the atom type pairs (86 out of 280) were classified in this category (see Table S1 in Additional file 1). All of these are known to exhibit specific interactions, such as hydrogen bonding, salt bridge, and polar-polar interactions. Examples of this category include hydrogen bonding pairs, such as NLB-O2A and NLB-OLC, which represent a hydrogen bonding interaction between a primary or secondary amine (NLB) and a double-bonded oxygen (O2A) or charged oxygen (OLC).

For the second category (Fig. 1b), the distribution curve increased rapidly over a short span of distance, then stayed fairly flat or decreased slowly over a wide distance range. This type of distribution is indicative of the absence of a strongly preferred distance for the two atom types to contact each other. About half of the atom type pairs (137/280) fell into this category. Most were for weak interactions (80/137), such as C3N-C3N (two non-polar sp3 carbon atoms) and CRN-CRN (two non-polar aromatic carbons), or for less well-defined interactions (57/137) involving sulfuric or aromatic atom types, such as S3N-CRP (a sp3 sulfur atom that is usually non-polar, but may be deprotonated on rare occasions, interacting with a polar aromatic carbon) and NRD-CRN (a hydrogen bond donor nitrogen interacting with a non-polar aromatic carbon).

The distribution curve of the third category (Fig. 1c) also began to rise after a short distance, but, unlike the first two categories, rose quite slowly over a large distance. This indicates that the atom type pairs belonging to this category rarely see each other at short distances. The few occurrences observed at short distances are likely to be a result of an attraction owing to adjacent atoms that are chemical-bonded to the atom in question. Thirty-eight atom type pairs were classified into this category, which typically exhibited an interaction between a non-polar atom and a polar atom or between two polar atom types with the same polarity, such as C2N-C2P (a non-polar sp2 carbon interacting with a polar sp2 carbon) and NLC-NRD (a charged nitrogen interacting with a hydrogen bond donor nitrogen).

The fourth category (Fig. 1d) showed a noisy curve that did not display an easily identifiable pattern, which may indicate insufficient data, as they usually contained an atom type of rare occurrence, such as halogen in HAL-NLB and metal in NLC-MET. There were 19 atom type pairs in this category.

A common observation for the first three categories of atom type pairs was a zero occurrence when the distance was short enough, suggesting that a distance threshold could be established and that only above this could attraction overcome repulsion, allowing the pair to be observed. In addition, the occurrence distribution curves of the first two categories fell after reaching a peak, displaying a specific upper threshold. The third category did not need an upper threshold, since its atom type pairs preferred not to contact each other. For the fourth category, we decided to use 4Å and 2Å as the upper and lower threshold values, which are arbitrary choices, but are within the range determined for the other three categories of interaction. For other rarely observed atoms, such as boron, that are not defined in our simple set of 23 atom types (Table 1), the arbitrarily chosen thresholds of the fourth category along with the associated network motifs can be applied to them to facilitate subsequent computations.

Despite the common main features, these occurrence distribution curves were not smooth, making an automated determination of thresholds difficult, so we manually inspected the data and recorded the lower and upper thresholds for each of the 280 atom type pairs. In all, the upper thresholds thus determined ranged from 3Å to 5.6Å and the lower thresholds from 1.2Å to 4Å. (see Table S1 in Additional file 1).

### Construction of protein-ligand interaction networks

**Figure 2:**
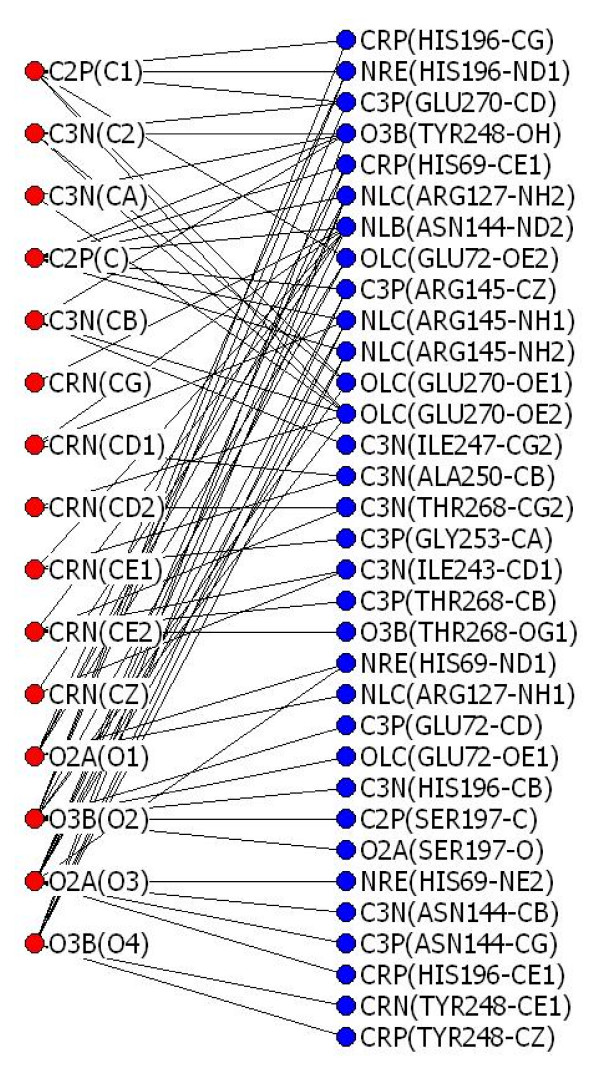
**A protein-ligand interaction network**. This example shows the protein-ligand interaction network transformed from the 3D coordinates of the complex of carboxypeptidase A and l-benzylsuccinate (PDB entry 1cbx). Each ligand atom in this network is represented by a combination of a **red **circle, its 3-code atom type, and its atom name from the PDB file in the parenthesis. Likewise, each protein atom is represented by a combination of a **blue **circle, its 3-code atom type and, in parentheses, its residue name, residue number, and atom name from the PDB file. Each black line connects a ligand atom and a protein atom for which connectivity (interaction) has been established based on distance thresholds.

With the upper and lower thresholds for all the interactions determined, we then constructed protein-ligand interaction networks using the following procedures: 1) carrying out atom type assignment on the atoms of the protein and ligand (hydrogen atoms were not considered in this work), 2) checking all the protein-ligand interatomic distances against the distance thresholds of the corresponding atom type pairs, and 3) connecting those that met the thresholds and ignoring those that did not. As an example, Fig. 2 shows a 2D representation of the 3D protein-ligand interaction network constructed for the complex of carboxypeptidase A and l-benzylsuccinate (PDB entry 1cbx).

### Motif searching and development of *MotifScore*

The next step was to identify motifs from the total of 6,276 protein-ligand interaction networks constructed as described above. While a number of algorithms for discovering network motifs have been reported [28-32], they could not be easily adopted here because the protein-ligand interaction networks constructed in this study are a bipartite network, which is different from the conventional gene-gene or protein-protein interaction networks, in which there is usually only one type of node and for which the existing motif identification algorithms have usually been developed. Moreover, since there were only 23 atom types, a number much smaller than the number of interacting atoms, many nodes within the same protein-ligand network were indistinguishable, which is quite different from the situation in other biological networks, where a node usually does not occur more than once in the same network.

In the total of 6,276 protein-ligand interaction networks constructed, the largest number of protein atoms connected to a ligand atom was 17. Thus, a network motif involving just one ligand atom would be those connecting 1 (ligand atom) to 2 (protein atoms), 1 to 3,..., up to 1 to 17. However, mathematically, of these "1 interacting with n" motifs, the number of those connecting to 7 or 8 protein atoms would be the largest (i.e.  and  >> all other ). In the actual data, the 1 interacting with 7 and 1 interacting with 8 motifs indeed dominated the 1 interacting with n motifs (data not shown), and, because the number of 1 interacting with 7 and 1 interacting with 8 motifs was so huge, the other types of motifs became negligible and a scoring function based on these would be severely biased to the 1 interacting with 7 and 1 interacting with 8 motifs. Consequently, we decided to search for other simple motifs, such as those of 2 ligand atoms interacting with 3 protein atoms, where the scoring function would not be severely biased to a particular type of motif.

**Figure 3:**
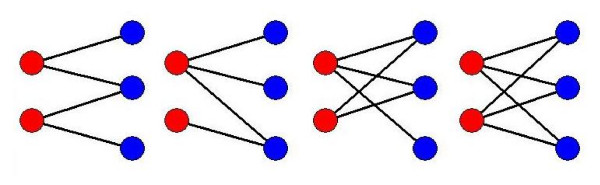
**Four distinctive types of simple motifs considered in this work**. The four have different connection topologies: 2 red nodes (ligand atoms) interacting with 3 blue nodes (protein atoms) with 4, 5, or 6 connections. The interactions are symbolized by the black lines.

There are four distinctive topologies of the 2 ligand atoms interacting with 3 protein atoms motifs, as shown in Fig. 3. In all, we obtained 395,240 such interaction motifs, the large number arising from the numerous combinations of the 23 atom types (Table 1) that could be assigned to the five constituent atoms.

For various reasons, other simple motifs were not included in *MotifScore*: the 1 interacting with 2 and 2 interacting with 1 motifs are likely to occur randomly, as they contain only two connections; there was a comparatively very small number of 3 (ligand atoms) interacting with 1, 3 interacting with 2, and 3 interacting with 3 motifs due to the small number of ligand atoms that can simultaneously interact with protein atoms under our interaction criteria; and, for the 2 interacting with 2 and 1 interacting with 3 motifs, no significant difference in the performance of *MotifScore *was seen when they were included (data not shown).

Intuitively, without the guidance of energy computation, a network motif-based scoring function stipulates that the presence of more motifs implicates better interactions (i.e. better docking solutions). However, in developing *MotifScore*, we immediately faced two major difficulties: 1) as mentioned, due to the large number of combinations of atom types, even the simple 2-3 network topologies (Fig. 3) harbored a large number of distinctive motifs which should not all be counted equally, because, for instance, some motifs consist of more frequently occurring atom types than others; 2) if the ligand molecule was pushed into the binding site to make more contacts with the protein, new motifs would be created and some of the old motifs removed, but the rate of increase would far outweigh the rate of loss. To overcome these difficulties, *MotifScore *was consequently made a composite of two opposing components, *Gain *and *Penalty*, with *Gain *being a sum of the normalized motif counts and *Penalty *being a factor using number of clashes between ligand and protein atoms to avoid motif overcounting, as described below.

In Eq. 1 we define, for each of the total 395,240 motifs, a significance grade, *SG*, which reflects the relative importance of a specific motif in the network.(1)

where *OM_i _*denotes the occurrence of a specific motif *i *in the 6,276 protein-ligand interaction networks constructed from the complex structures in the PDB; motif *i *is one of 395,240 motifs, each of which consists of 5 atoms, 2 on the ligand and 3 on the protein, and of 4-6 network edges, depending on which of the four motif types (Fig. 3) motif *i *belongs to; *N_i _*is the number of motifs having the same motif type as motif *i*; *OA_j _*denotes the occurrence of atom type *j *(one of 14 protein atom types or 20 ligand atom types; see Table 1) in the 6,276 networks, where atom type *j *(*j *= 1,5) is the atom type assigned to one of the 5 atoms constituting motif *i*; and *M_j _*is either *M_l _*or *M_p _*(*M_l _*if atom type *j *is a ligand atom and *M_p _*if atom type *j *is a protein atom), where *M_l _*(*M_p_*) is the total number of ligand (protein) atoms.

Using normalization by the total occurrence of each motif type and the relative occurrence of each atom type, Eq. 1 takes into account the fact that motifs consisting of fewer edges or more prevalent atom types are likely to occur more often. With Eq. 1, a motif's *SG *would not necessarily increase in a larger dataset of protein-ligand complex structures in which motif occurrences are bound to increase. Because each motif's *SG *reflects the probability of the motif occurring in the interaction network, the probability of it occurring simultaneously with other motifs should be a multiplication of all their *SG*s. For easier computation, motif *SG *was log transformed to convert multiplication into addition.

To score a specific protein-ligand interaction network, i.e. a specific protein-ligand complex conformation, the *Gain *component (Eq. 2) of *MotifScore *is the sum of the motif *SG *(Eq. 1) for all the motifs found in this specific network, or the particular protein-ligand binding conformation from which the network resulted.(2)

where *SG_k _*is the motif significance grade (Eq. 1) for the *k^th ^*motif found in the specific network constructed from a specific docking solution, and *n *is the total number of motifs present in this network.

As mentioned earlier, we need a penalty to counteract the excessive motif gains of a growing interaction network that may occur in docking when one places the ligand molecule closer and closer to its protein receptor.(3)

In Eq.3, *NC *denotes the total number of clashes between ligand and protein atoms, where one clash is counted when the distance between two atoms, one each from the protein and ligand, is less than the lower threshold of their atom type pair (see Fig. 1) and weight *W *is a parameter to be optimized against a subset of LPDB, which served as a training set, as described above. The value of 10 for *W *appeared to yield the best result, though the results were not very sensitive to the value of this parameter (Fig. S1 in Additional file 1). A second parameter was also introduced to adjust the lower distance thresholds of the interacting atom type pairs for best fit of the training data. As described earlier, the lower thresholds were determined based on the observed distribution of interatomic distances. However, at a distance barely exceeding the lower threshold, the probability of observing the atom type pairs in the native structures is rather low, whereas, when evaluating the conformations produced in the docking process, for any distance that was above the lower threshold, even just barely, a connection/interaction would be made and it would contribute to motif *Gain*. In order to reflect this reality better, we modified the lower distance thresholds by a factor and found that better results could be obtained when the lower distance thresholds for computing the *Gain *component were raised by 10 percent compared to those used to compute *Penalties *(Fig. S2 in Additional file 1), though, like the *Penalty *weight *W *(Fig. S1 in Additional file 1), the results were not very sensitive to the value of the lower threshold factor used (Fig. S2 in Additional file 1).

Finally, after experimenting with several different composite formulae, we settled on one with a ratio for *MotifScore *(Eq. 4), which yielded the best results.(4)

## Results and Discussion

### Scoring docking solutions

As in many related studies, we evaluated the performance of *MotifScore *using the criteria of distance rmsd (root-mean-square-deviation) between the experimentally observed ligand positions and those of the highest-scored docking solution to consider whether the scoring was a success or a failure. We used two docking datasets, one by Brooks et al. [8] for parameter optimization (Fig S1,S2 in Additional file 1) and the other by Wang et al. [9] for performance comparison with 12 other scoring functions (Table 2). Our success rate using the criterion of rmsd ≦2 Å was 84% for both the parameter optimization set (Fig. S1 and S2 in Additional file 1) and Wang's benchmark test set (Table 2). As shown in Table 2, compared to other scoring functions, *MotifScore *performed admirably, being second only to DrugScore^CSD ^[20]. DrugScore^CSD ^is a significantly improved version of DrugScore^PDB ^[19]. DrugScore^PDB ^was developed based on the PDB and DrugScore^CSD ^was developed based on the Cambridge Structural Database (CSD) [33], which is a higher-resolution data source for contact distances between interacting atoms, a difference thought to underlie the improvement [20]. Whether *MotifScore *can be similarly improved using the CSD instead of the PDB remains to be determined, but the effect may not be as significant as in DrugScore^CSD^, as it is likely that the network motif-based scoring (Eq. 1-4) cannot be fine-tuned as much as a conventional energy-based scoring function, such as that used by DrugScore^CSD^. Nevertheless, it is encouraging that, despite its present crude form, *MotifScore *performed surprisingly well and therefore can serve as a non-conventional alternative to existing scoring functions, being useful especially for coarse-grained docking computations.

**Table 2:** Docking scoring success rates for different scoring functions.^a^

Scoring function	Success rate (%) using different rmsd criteria^b^		
	
	≦1 Å	≦2 Å	≦3 Å
DrugScore^CSD^	83	87	*^c^
***MotifScore***	71	84	86
Cerius2/PLP	63	76	80
SYBYL/F-Score	56	74	77
Cerius2/LigScore	64	74	76
DrugScore^PDB^	63	72	74
Cerius2/LUDI	43	67	67
X-Score	37	66	74
AutoDock	34	62	72
Cerius2/PMF	40	52	57
SYBYL/G-Score	24	42	56
SYBYL/ChemScore	12	35	40
SYBYL/D-Score	8	26	41

^a ^The data were taken from Wang et al. [9] and Velec et al. [20], except for the *MotifScore *results, which were computed in this work using the same dataset.

^b^Scoring functions are ranked by their success rates at rmsd ≦ 2.0 Å (the success rate of a scoring function is calculated by checking if the rmsd value of the highest-scored conformation is less than, or equal to, the specified rmsd criterion from the experimentally observed conformation.)

^c^Not provided by Velec et al. [20]

The 100-complex dataset of Wang et al. [9] had also been divided into subsets of hydrophilic, mixed, and hydrophobic complexes to evaluate potential bias of scoring functions on different types of molecular interactions. As summarized in Table 3, *MotifScore *achieved a very high success rate of 91% on both the hydrophilic and mixed subsets, but, like many other scoring functions, including DrugScore^PDB ^(result for DrugScore^CSD ^not available), its performance was relatively poor for the hydrophobic subset. The less favorable result for the hydrophobic subset is thought to be due to several difficult cases in this subset in which the binding site is shallow and hard to score accurately [9], compounded by the fact that the number (24) of complexes in this subset is much smaller than in the other two subsets, which means that the success rate would drop by as much as 4% if the number of correctly predicted complexes was reduced by only one.

**Table 3:** Success rates of different scoring functions on subsets of different types of molecular interactions

Scoring function	Success rate (%)			
	
	Overall (100)	Hydrophilic (44)	Mixed (32)	Hydrophobic (24)
*MotifScore*	84	91	91	63
Cerius2/PLP	76	77	78	71
SYBYL/F-Score	74	75	75	71
Cerius2/LigScore	74	77	75	67
DrugScore^PDB^	72	73	81	58
Cerius2/LUDI	67	75	66	54
X-Score	66	82	59	46
AutoDock	62	73	53	54
Cerius2/PMF	52	68	44	33
SYBYL/G-Score	42	55	34	29
SYBYL/ChemScore	35	32	34	42
SYBYL/D-Score	26	23	28	29

The data were taken from Wang et al. [9], except for *MotifScore*. The success rates were determined using rmsd ≦ 2.0 Å.

### Some significant motifs

To elucidate which of the hundreds of thousands of motifs make the greatest contribution to *MotifScore*, we ranked them by their motif *SG*. The top 30 motifs are listed in Table 4. All contain aromatic and/or polar (including charged) atom types on the protein side. This agrees well with the observation that the frequently observed catalytic residues of proteins are generally the residues with aromatic, polar, or charged side-chains [34,35]. In addition, although cysteine occurs less often than other amino acids in proteins, it is relatively enriched in catalytic residues [35]. The sulfur atom of cysteines, assigned as S3N, was also commonly observed in our top-ranked motifs. Similarly, there is a relatively small number of halogens in protein binding ligands, but, when present, these atoms usually play a key role in protein-ligand interactions. The normalized motif count, or *SG*, reflects the significance of these halogens: atom type HAL-containing motifs appear several times in the top 30 motifs even though their occurrences are small.

**Table 4:** Top 30 protein-ligand interaction network motifs ranked by motif significance grades, *SG*.

Rank	Ligand atom types	Protein atom types	Occurrence	Significance grade	Motif type
1	HAL, HAL	CRN, S3N, S3N	31	11.79	5 connections
2	C2N, ORA	NRE, S3N, S3N	1	11.69	5 connections
3	HAL, HAL	S3N, S3N, S3N	6	11.67	4 connections, 2+2
4	C2N, ORA	CRP, S3N, S3N	1	10.89	5 connections
5	NRA, NRA	CRN, CRN, NRE	1597	10.71	Fully connected
6	HAL, HAL	NRE, O3B, S3N	5	10.62	5 connections
7	CRN, ORA	CRN, CRN, CRN	374	10.57	Fully connected
8	C2N, ORA	S3N, S3N, NRE	1	10.55	4 connections, 2+2
9	CRN, NLC	CRN, CRN, CRN	2372	10.54	Fully connected
10	CRP, NRA	CRN, CRN, CRP	5236	10.49	Fully connected
11	C2N, ORA	NRE, S3N, NLB	1	10.40	5 connections
12	NRA, NRA	CRN, CRN, CRP	2572	10.40	Fully connected
13	CRP, NRA	CRN, CRP, CRP	1227	10.36	Fully connected
14	C2N, C2N	CRN, CRN, NRE	66	10.29	Fully connected
15	HAL, HAL	CRP, O3B, S3N	8	10.29	5 connections
16	HAL, NLC	CRN, S3N, S3N	25	10.28	4 connections, 3+1
17	NRA, NRA	CRN, NRE, NRE	125	10.28	Fully connected
18	NRA, NRA	CRN, CRP, NRE	259	10.22	Fully connected
19	HAL, HAL	S3N, CRN, S3N	27	10.20	4 connections, 3+1
20	CRN, NLC	CRN, CRN, CRP	450	10.20	Fully connected
21	HAL, HAL	CRN, O3B, S3N	2	10.17	Fully connected
22	CRN, CRN	CRN, CRN, CRN	37501	10.12	Fully connected
23	CRP, CRP	CRN, CRP, CRP	1676	10.05	Fully connected
24	HAL, HAL	S3N, NRE, S3N	2	10.03	4 connections, 2+2
25	HAL, NLA	S3N, S3N, NLC	2	10.01	5 connections
26	NRA, NRA	CRN, CRN, CRN	6605	10.01	Fully connected
27	HAL, NLC	CRN, S3N, NLB	50	10.00	4 connections, 2+2
28	HAL, HAL	CRN, S3N, S3N	14	9.86	4 connections, 2+2
29	HAL, HAL	CRN, CRN, CRN	68	9.86	Fully connected
30	HAL, NLC	CRN, S3N, NLC	50	9.82	4 connections, 2+2

The occurrences were counted in the 6,276 protein-ligand complex structures used to construct the networks (see Methods). See Fig. 3 for the different motif types.

**Figure 4:**
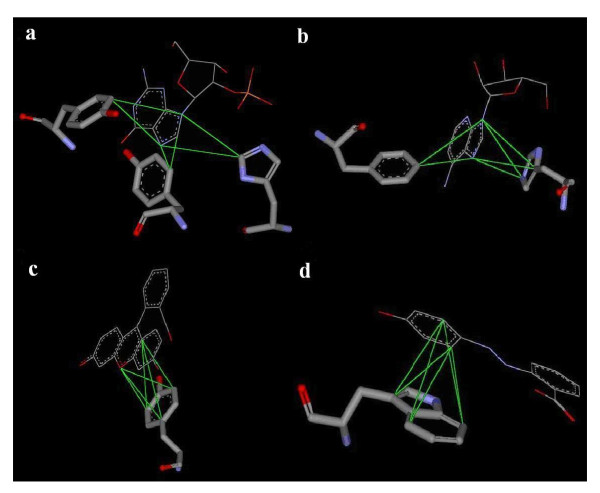
**Four significant motifs involving interactions between aromatic rings**. Ligand molecules are shown as line model and protein side-chains as stick model. The green lines show the connectivities (edges) of the motifs, which, in these examples, are a 2 (ligand atoms) interacting with 3 (protein atoms) fully connected motif. (**a**) (NRA, NRA) interacting with (CRN, CRN, CRP): 2 ligand atoms interacting with 3 protein atoms located on 3 different aromatic residues; (**b**) (NRA, NRA) interacting with (CRN, CRP, NRE): 3 protein atoms located on 2 aromatic residues and the aromatic ring of histidine is parallel to that of adenine in the ligand; (**c**) (CRN, ORA) interacting with (CRN, CRN, CRN): all 3 protein atoms located on 1 residue, tyrosine; (**d**) (CRN, CRN) interacting with (CRN, CRN, CRN): this motif is the most prevalent motif (in number) and is ranked 22^nd ^(by significance grade) in Table 4.

Fig. 4a-d illustrate four of the top 30 ranked motifs observed in the 6,276 protein-ligand complexes. The four examples all exhibit interactions between aromatic atom types. As can be seen, these motifs are a composite of multiple pairwise interactions between two ligand interacting sites (atoms) and three protein atoms from one (**c **and **d**), two (**b**), or three (**a**) amino acid residues. Interestingly, in Fig. 4b-d, the aromatic rings of the ligand and of the amino acid side-chains stack against each other, formingπ-πinteractions [36]. These ring-stacking interactions are not easily modeled by conventional scoring functions using separate accounts of pair-wise interactions, but, collectively, they emerge as significant motifs in protein-ligand interaction networks.

### Binding site-enriched protein triangles

Since all of the protein-ligand interaction motifs in our model contain 3 protein atoms, we wondered whether the chance of observing three spatially close protein atoms simultaneously was quite different in the ligand-binding site compared to the rest of the protein. To answer this question, we computed a binding site enrichment factor, *F_b_*, using Eq. 5, for every distinguishable group of three protein atom types captured in our 2 interacting with 3 ligand-protein interaction motifs. For convenience, we called the three protein atoms a protein triangle. Note that, as protein triangles are distinguished by the atom types assigned to their three constituent atoms, they are atom type triangles.(5)

Eq. 5 was adopted here based on the work of Zeeberg et. al. [37], where *n_s _*and *N_s _*are the occurrences of a specific atom type triangle found, in, respectively, the interaction network and the whole protein and *n *and *N *are the corresponding total number of atom type triangles. To count the triangles not only in the binding site, but also in the whole protein, we searched for those with all three sides longer than 2 Å and two less than 10 Å and the other less than 13 Å, which would account for almost all (>99.5%) of the triangles present in the protein-ligand interaction motifs identified. Theoretically, with 14 protein atom types and taking repetition into account, we should have a total of 560 different atom type triangles. However, as metal ions rarely occur in our dataset of protein structures and almost all of those that do are involved in ligand binding, the *F_b_*s for metal-containing triangles were extremely large. They were therefore excluded in the discussion below, leaving 455 non-metal triangles to be ranked and sorted by their *F_b _*(see Table S2 in Additional file 1).

**Figure 5:**
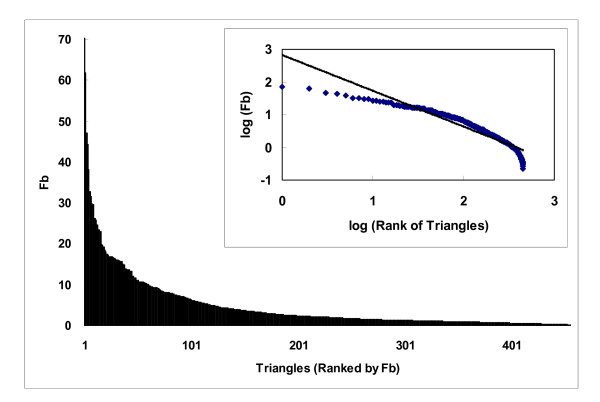
**Uneven distribution of the binding-site enrichment factors (*F_b_*) for 455 protein atom type triangles**. The insert shows the fitting of the distribution to a power-law expression, y~x^-γ^, withγestimated to be 1.095 (r^2 ^= 0.89) for the best fit.

**Figure 6:**
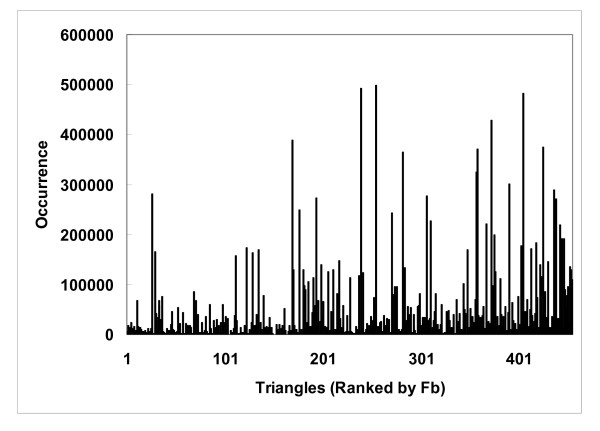
**Occurrences of protein atom type triangles in protein-ligand interaction networks (i.e. ligand binding sites) do not correlate well with binding site enrichment factors**. The protein atom type triangles are ranked by the binding site enrichment factor *F_b_*.

**Figure 7:**
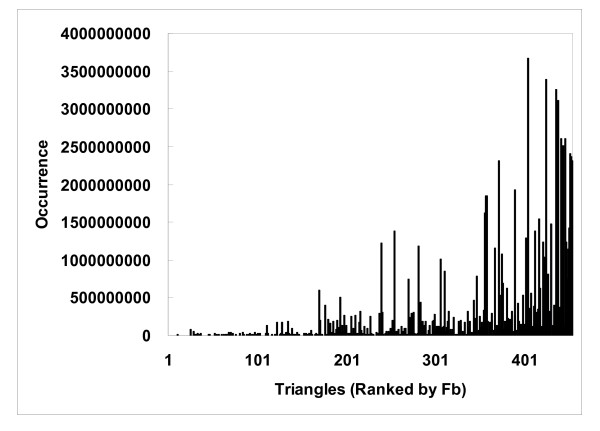
**Occurrences of protein atom type triangles in the whole protein show trend with binding site enrichment factors**. The protein atom type triangles are ranked by the binding site enrichment factor *F_b_*.

**Figure 8:**
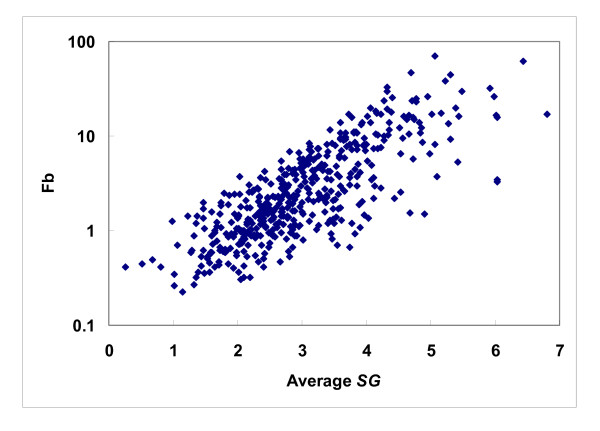
**Binding site enrichment versus significant grade**. The scatterplot shows the relationship between the binding site enrichment factor *F_b _*for protein triangles versus the average significance grade (*SG*, Eq. 1) for the triangle-containing motifs. The correlation between the two has a Spearman correlation coefficient value of 0.78.

The binding site enrichment factor *F_b _*represents a protein triangle's propensity for occurring in the protein-ligand interaction networks constructed, or, in other words, the ligand-binding sites. As shown in Fig. 5, such propensity is far from uniform, and, in fact, the distribution resembles that observed for a wide spectrum of biological properties: the distribution of *F_b _*tended to follow a power law distribution [38], y~x^-γ^(γwas estimated to be 1.095, r^2 ^= 0.89), suggesting that only a handful of triangles were highly enriched in ligand-binding sites, while most showed little or no propensity [223/455 (49%) had an *F_b _*below 2 and 101 (22%) below 1]. Some low frequency triangles appear to be concentrated at ligand-binding sites. For example, the NRE-NRE-NRE triangles, which are triangles that connect 2 or 3 different histidines (there were few NREs from tryptophan in our motifs), were not commonly observed, but, when they were, they tended to be at ligand-binding sites (their *F_b _*was 17.3). The uneven distribution of *F_b _*(Fig. 5), which was normalized to account for the wide range of occurrences of these triangles (Eq. 5), suggests that *F_b _*does not necessarily correlate with binding site occurrence (Fig. 6), although triangles with a high *F_b _*tended to occur less frequently in other parts of the protein (Fig. 7). Many of the highly enriched triangles were constituents of the top-ranked motifs (Table 4, 5) that were major contributors to the *MotifScore*. Consequently, as can be seen from Fig. 8, triangles with higher *F_b _*values also tended to form motifs with a higher *SG *(Eq. 1); the correlation between the two was significant, with the Spearman correlation coefficient being 0.78.

Interestingly, Table 5 shows that some of the binding-site enriched protein triangles consisted of 3 charged or polar atom types with the same positive (or partially positive) or negative (or partially negative) charge. For example, the most enriched triangle consisted of 3 NLCs, which are nitrogen atoms with a positive charge on lysine or arginine residues. The reason why these positively charged amino acids, which are spatially close, as they form the triangle, are enriched in the active site is probably because they all interact with the ligand molecule at the same time.

**Table 5:** Top 30 protein atom type triangles ranked by their binding site enrichment factor *F_b_*.

Rank	Triangle	*F_b_*	Coverage%* (binding site)	Coverage%* (whole protein)
1	NLC-NLC-NLC	70.6	13.2	93.7
2	CRP-CRP-CRP	61.9	23.3	96.6
3	CRP-NLC-NLC	47.2	16.6	98.0
4	CRP-CRP-NLC	44.3	20.1	97.7
5	CRP-CRP-OLC	38.1	22.6	98.3
6	NLC-NLC-NRE	32.8	8.2	95.7
7	CRP-CRP-NRE	31.7	16.8	93.6
8	CRP-CRP-O3B	29.8	26.4	98.2
9	NLC-NLC-O3B	29.6	16.3	98.6
10	CRP-CRP-NLB	26.2	19.8	98.1
11	CRN-CRP-CRP	26.2	32.4	98.5
12	CRP-OLC-OLC	25.8	21.0	98.8
13	NLB-NLC-NLC	24.6	20.3	98.9
14	CRP-NRE-OLC	23.5	15.4	97.6
15	CRP-NLC-O3B	23.5	19.1	98.3
16	CRP-NLC-NRE	23.0	12.1	96.9
17	NRE-OLC-OLC	19.9	12.5	97.9
18	CRP-NRE-NRE	19.7	9.0	89.4
19	CRP-NLB-NLC	19.2	17.2	98.3
20	NLC-O3B-O3B	18.6	14.2	98.5
21	NRE-NRE-OLC	18.1	8.3	96.7
22	CRN-NLC-NLC	17.5	17.4	98.6
23	NRE-NRE-NRE	17.3	4.2	77.3
24	OLC-OLC-OLC	17.0	13.0	98.4
25	CRP-NLC-OLC	17.0	15.9	98.3
26	CRN-CRN-CRN	16.9	41.4	99.0
27	CRP-CRP-S3N	16.8	5.3	93.6
28	NLC-NRE-NRE	16.7	6.1	94.2
29	CRN-CRN-CRP	16.5	37.6	98.9
30	C3P-NLC-NLC	16.2	33.5	98.9

*Coverage: The percentage of protein-ligand complexes (out of the 6,276 complexes) in which a specific triangle was present in the ligand-binding site or the whole protein.

As shown in Table 5, the top 30 binding site-enriched triangles were generally composed of atom types (namely, CRP, NRE, NLC, NLB, OLC, O3B, and S3N) that exist on the side-chains of the amino acids histidine, lysine, arginine, asparagine, aspartic acid, glutamic acid, glutamine, serine, tyrosine, threonine, and cysteine. A survey by Barlett et. al. [34] found that, of the 20 amino acids, only these 11 polar and charged residues are directly involved in catalytic reactions. Gutteridege and Thornton [35] further proposed that histidine is the most commonly observed and most important residue in protein enzymatic reactions, followed by, in descending order of observance, cysteine, the charged residues glutamate, aspartate, arginine, and lysine, and, finally, the polar residues serine, threonine, tyrosine, glutamine, and asparagine. This is fairly consistent with our statistics on the ligand-binding site enrichment: Of the 30 protein triangles that were most enriched in ligand-binding sites, CRP (on residues with polar and aromatic side chain, including histidine) was the most commonly observed atom type, while NLC (on lysine or arginine), NRE (primarily on histidine), and OLC (on aspartic acid or glutamic acid) were also frequently observed, and NLB (on glutamine or asparagines) and O3B (on serine, threonine, or tyrosine) on polar residues were observed less often. Unlike in Table 4, S3N (on cysteine) appeared only once in Table 5. This can be explained by the fact that whereas every protein triangle in Table 5 is unique, the same triangle can appear multiple times in Table 4 together with different interacting ligand atoms and/or in different motif topologies (Fig. 3).

Many of the binding site-enriched triangles are composites of multiple residues. For example, many of the NRE-NRE-OLC triangles were composed of two histidines and a carboxylate residue, corresponding to the 2-His-1-carboxylate facial triad, a characteristic motif of a metalloenzyme superfamily [39]. Similarly, many of the NRE-NRE-NRE triangles were composed of three histidines, known as the histidine triad of the nucleotide-binding histidine triad superfamily [40,41]. Interestingly, while only 28 and 2 protein structures were annotated by PDB [23] as having, respectively, a histidine-triad or a 2-His-1-carboxylate facial triad in the 6,276 protein-ligand complex structures we analyzed, roughly 2% and 5% of these proteins contain NRE-NRE-NRE (on 3 different histidines) and NRE-NRE-OLC (on 2 histidines and an aspartic/glutamic acid) atom type triangles in their ligand-binding sites, respectively. Although these proteins may not be members of the histidine triad superfamily or the 2-His-1-carboxylate facial triad superfamily per se, the much higher prevalence than previously recognized of these motifs may guide future investigations to identify conserved catalytic mechanisms in diverse enzyme families.

### Issues and prospects of *MotifScore*

Although *MotifScore *has been derived using static crystallographic structures of protein-ligand complexes, the use of non-precise interaction distances and counts of number of motifs instead of interaction energies likely renders it more tolerant to subtle conformational changes in protein or ligand than are conventional energy-based scoring functions, which are known to be sensitive to steric clashes especially in unbound dockings [7,42]. The results on both the LPDB and Wang's dataset attest the ability of *MotifScore *to account for at least ligand flexibilities, since the ligands, though not the proteins, in the decoys of both sets include flexible conformations generated by molecular dynamics simulation and genetic algorithm, respectively. Nevertheless, the performance of *MotifScore *in dockings that sample different protein conformations (e.g. [42]) requires further examinations.

*MotifScore *can be regarded as a kind of knowledge-based scoring function since it was also derived by extracting information from a statistical analysis of known protein-ligand complex structures. However, *MotifScore *is different from conventional knowledge-based scoring functions such as PMF and DrugScore [18-20] in at least two aspects: 1) it is non-energy based, and 2) using the interaction network motifs, it can score directly on the 3D interaction patterns of molecular recognition conserved in protein ligand complexes. One disadvantage of *MotifScore*, however, is that a non-conventional search scheme may need to be developed to take advantages of its unique features, as discussed next.

**Figure 9:**
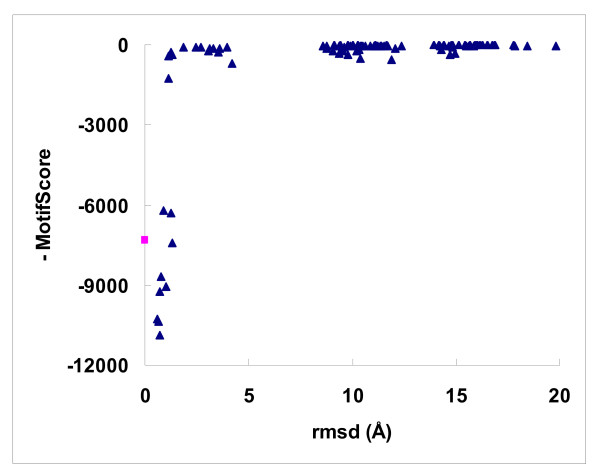
**The lanscape of *MotifScore***. The scatterplot shows the landscape of *MotifScore *values for a typical success case (PDB code 1APB). The 100 docking solutions (decoys), marked by triangles, were taken from Wang's dataset and the square represents the native complex conformation.

As *MotifScore *is non-energy-based, it was of interest to examine the landscape of its functional values. We found that *MotifScore *did not correlate well with experimentally determined binding affinities, the Spearmen correlation coefficient between the two being only 0.259, only better than that (0.141) of AutoDock among the various scoring functions evaluated by Wang et al. [9]. Nevertheless, Fig. 9 shows that, for a typical success case, *MotifScore *could easily distinguish reasonably good docking solutions (rmsd < 2 Å) from bad ones. Intriguingly, there appears to be a very narrow funnel leading to the native state formed by very good docking solutions. This is quite distinct from that of a conventional energy-based scoring function where the funnel leading to the energy minimum, which should be reasonably close to the native state, is usually much smoother [43]. This implies that, whereas a search algorithm, such as a genetic algorithm, may work efficiently with a conventional, energy-based scoring function to find good docking solutions, its direct adoption for use with *MotifScore *would not be ideal. On the other hand, the protein-ligand interaction motifs derived in this work, in their three dimensional arrangements of interacting atoms, capture the spatial arrangements of reasonably good docking solutions, so it is quite possible to develop a scheme to look up the table of interaction network motifs (e.g. Table 4) and directly home in on a reasonable docking solution based on the structure of a few top-ranked motifs. This could eliminate the time-consuming searching computation needed in conventional docking methods, or at least provide a good starting solution for further refinement. In addition, although *MotifScore *is currently limited to scoring the interactions between protein and small molecules, the same network-based approach should be extendable to protein-protein and protein-DNA/RNA interactions, which form conserved spatial and chemical binding patterns (e.g., [44,45]). Work along these lines is currently undergoing in our laboratory.

## Conclusions

*MotifScore *is a novel interaction-motif-based scoring function for protein-ligand docking. Despite the absence of mathematical models to mimic the force field of molecular interactions, *MotifScore *performed well in distinguishing between good and bad docking solutions in a benchmark test set. Furthermore, owing to the network approach, *MotifScore *is intrinsically more able than conventional docking scoring functions to capture interactions involving more than two interacting sites, the π-π stacking of two aromatic rings being a prime example. The ligand-binding site-enriched interaction motifs identified are in accord with existing knowledge on protein-ligand binding and may prove useful for binding site predictions. Finally, the three-dimensional protein-ligand interacting motifs could provide very good templates for placing ligand molecules in fast, though coarse, protein docking computations.

## Availability and requirements

The source code of *MotifScore *is available for download at: https://sourceforge.net/projects/msdock/. The package contains a set of Perl scripts for computing the scoring function and for creating a Perl database file that stores the names of ligands and their atom types. It also offers some demonstration examples of how to obtain the final docking scores. These scripts work on a Unix or Linux platform and their download is free for academic users.

## Authors' contributions

ZRX carried out the research and drafted the manuscript. MJH conceived the project, guided the research, and revised the manuscript. Both authors read and approved the final manuscript.

## Supplementary Material

Additional file 1 **Supplementary data for details of motif related parameters**. This file contains 2 tables and 3 figures. Table S1 lists the lower and upper distance thresholds for each of the 280 atom type pairs. Table S2 lists the binding site enrichment factor, Fb, of each of the 455 non-metal protein atom type triangles. Figs. S1 and S2 display the optimization results of two parameters, *Penalty *weight *W *and the raising percentage of lower thresholds, on the training dataset LPDB. Fig. S3 shows a schematic presentation for some of the atom types defined in Table 1. Click here for file

## References

[B1] Cummings MD DesJarlais RL Gibbs AC Mohan V Jaeger EP Comparison of automated docking programs as virtual screening tools J Med Chem 2005 11 4 962 976 10.1021/jm049798d 15715466

[B2] Grzybowski BA Ishchenko AV Shimada J Shakhnovich EI From knowledge-based potentials to combinatorial lead design in silico Acc Chem Res 2002 11 5 261 269 10.1021/ar970146b 12020163

[B3] Schneider G Bohm HJ Virtual screening and fast automated docking methods Drug Discov Today 2002 11 1 64 70 10.1016/S1359-6446(01)02091-8 11790605

[B4] Taylor RD Jewsbury PJ Essex JW A review of protein-small molecule docking methods J Comput Aided Mol Des 2002 11 3 151 166 10.1023/A:1020155510718 12363215

[B5] Warren GL Andrews CW Capelli AM Clarke B LaLonde J Lambert MH Lindvall M Nevins N Semus SF Senger S Tedesco G Wall ID Woolven JM Peishoff CE Head MS A critical assessment of docking programs and scoring functions J Med Chem 2006 11 20 5912 5931 10.1021/jm050362n 17004707

[B6] Kitchen DB Decornez H Furr JR Bajorath J Docking and scoring in virtual screening for drug discovery: methods and applications Nat Rev Drug Discov 2004 11 11 935 949 10.1038/nrd1549 15520816

[B7] Halperin I Ma B Wolfson H Nussinov R Principles of docking: An overview of search algorithms and a guide to scoring functions Proteins 2002 11 4 409 443 10.1002/prot.10115 12001221

[B8] Ferrara P Gohlke H Price DJ Klebe G Brooks CL Assessing scoring functions for protein-ligand interactions J Med Chem 2004 11 12 3032 3047 10.1021/jm030489h 15163185

[B9] Wang R Lu Y Wang S Comparative evaluation of 11 scoring functions for molecular docking J Med Chem 2003 11 12 2287 2303 10.1021/jm0203783 12773034

[B10] Jones G Willett P Glen RC Leach AR Taylor R Development and validation of a genetic algorithm for flexible docking J Mol Biol 1997 11 3 727 748 10.1006/jmbi.1996.0897 9126849

[B11] Jones G Willett P Glen RC Molecular recognition of receptor sites using a genetic algorithm with a description of desolvation J Mol Biol 1995 11 1 43 53 10.1016/S0022-2836(95)80037-9 7823319

[B12] Morris GM Goodsell DS Halliday RS Huey R Hart WE Belew RK Olson AJ Automated docking using a Lamarckian genetic algorithm and an empirical binding free energy function J Comput Chem 1998 11 1639 1662 10.1002/(SICI)1096-987X(19981115)19:14<1639::AID-JCC10>3.0.CO;2-B

[B13] Kramer B Rarey M Lengauer T Evaluation of the FLEXX incremental construction algorithm for protein-ligand docking Proteins 1999 11 2 228 241 10.1002/(SICI)1097-0134(19991101)37:2<228::AID-PROT8>3.0.CO;2-8 10584068

[B14] Weiner PK Kollman PA AMBER--assisted model building with energy refinement--a general program for modeling molecules and their interactions J Comput Chem 1981 11 287 303 10.1002/jcc.540020311

[B15] Rarey M Kramer B Lengauer T Klebe G A fast flexible docking method using an incremental construction algorithm J Mol Biol 1996 11 3 470 489 10.1006/jmbi.1996.0477 8780787

[B16] Eldridge MD Murray CW Auton TR Paolini GV Mee RP Empirical scoring functions: I. The development of a fast empirical scoring function to estimate the binding affinity of ligands in receptor complexes J Comput Aided Mol Des 1997 11 5 425 445 10.1023/A:1007996124545 9385547

[B17] Sousa SF Fernandes PA Ramos MJ Protein-ligand docking: current status and future challenges Proteins 2006 11 1 15 26 10.1002/prot.21082 16862531

[B18] Muegge I Martin YC A general and fast scoring function for protein-ligand interactions: a simplified potential approach J Med Chem 1999 11 5 791 804 10.1021/jm980536j 10072678

[B19] Gohlke H Hendlich M Klebe G Knowledge-based scoring function to predict protein-ligand interactions J Mol Biol 2000 11 2 337 356 10.1006/jmbi.1999.3371 10623530

[B20] Velec HF Gohlke H Klebe G DrugScore(CSD)-knowledge-based scoring function derived from small molecule crystal data with superior recognition rate of near-native ligand poses and better affinity prediction J Med Chem 2005 11 20 6296 6303 10.1021/jm050436v 16190756

[B21] Leach AR Shoichet BK Peishoff CE Prediction of protein-ligand interactions. Docking and scoring: successes and gaps J Med Chem 2006 11 20 5851 5855 10.1021/jm060999m 17004700

[B22] Tirado-Rives J Jorgensen WL Contribution of conformer focusing to the uncertainty in predicting free energies for protein-ligand binding J Med Chem 2006 11 20 5880 5884 10.1021/jm060763i 17004703

[B23] Berman HM Bhat TN Bourne PE Feng Z Gilliland G Weissig H Westbrook J The Protein Data Bank and the challenge of structural genomics Nat Struct Biol 2000 11 Suppl 957 959 10.1038/80734 11103999

[B24] Roche O Kiyama R Brooks CL Ligand-protein database: linking protein-ligand complex structures to binding data J Med Chem 2001 11 22 3592 3598 10.1021/jm000467k 11606123

[B25] Ruvinsky AM Kozintsev AV The key role of atom types, reference states, and interaction cutoff radii in the knowledge-based method: new variational approach Proteins 2005 11 4 845 851 10.1002/prot.20385 15651026

[B26] Shulman-Peleg A Shatsky M Nussinov R Wolfson HJ Spatial chemical conservation of hot spot interactions in protein-protein complexes BMC Biol 2007 11 43 10.1186/1741-7007-5-43 17925020 PMC2231411

[B27] Soper AK The radial distribution functions of water and ice from 673 K and at pressures up to 400 MPa Chemical Physics 2000 11 121 137 10.1016/S0301-0104(00)00179-8

[B28] Babu MM Luscombe NM Aravind L Gerstein M Teichmann SA Structure and evolution of transcriptional regulatory networks Curr Opin Struct Biol 2004 11 3 283 291 10.1016/j.sbi.2004.05.004 15193307

[B29] Dobrin R Beg QK Barabasi AL Oltvai ZN Aggregation of topological motifs in the Escherichia coli transcriptional regulatory network BMC Bioinformatics 2004 11 10 10.1186/1471-2105-5-10 15018656 PMC357809

[B30] Koyuturk M Grama A Szpankowski W An efficient algorithm for detecting frequent subgraphs in biological networks Bioinformatics 2004 11 Suppl 1 i200 207 10.1093/bioinformatics/bth919 15262800

[B31] Milo R Shen-Orr S Itzkovitz S Kashtan N Chklovskii D Alon U Network motifs: simple building blocks of complex networks Science 2002 11 5594 824 827 10.1126/science.298.5594.824 12399590

[B32] Shen-Orr SS Milo R Mangan S Alon U Network motifs in the transcriptional regulation network of Escherichia coli Nat Genet 2002 11 1 64 68 10.1038/ng881 11967538

[B33] Taylor R Life-science applications of the Cambridge Structural Database Acta Crystallogr D Biol Crystallogr 2002 11 Pt 6 No 1 879 888 10.1107/S090744490200358X 12037325

[B34] Bartlett GJ Porter CT Borkakoti N Thornton JM Analysis of catalytic residues in enzyme active sites J Mol Biol 2002 11 1 105 121 10.1016/S0022-2836(02)01036-7 12421562

[B35] Gutteridge A Thornton JM Understanding nature's catalytic toolkit Trends Biochem Sci 2005 11 11 622 629 10.1016/j.tibs.2005.09.006 16214343

[B36] Petitjean A Khoury RG Kyritsakas N Lehn JM Dynamic devices. Shape switching and substrate binding in ion-controlled nanomechanical molecular tweezers J Am Chem Soc 2004 11 21 6637 6647 10.1021/ja031915r 15161291

[B37] Zeeberg BR Feng W Wang G Wang MD Fojo AT Sunshine M Narasimhan S Kane DW Reinhold WC Lababidi S Bussey KJ Riss J Barrett JC Weinstein JN GoMiner: a resource for biological interpretation of genomic and proteomic data Genome Biol 2003 11 4 R28 10.1186/gb-2003-4-4-r28 12702209 PMC154579

[B38] Liu WC Lin WH Davis AJ Jordan F Yang HT Hwang MJ A network perspective on the topological importance of enzymes and their phylogenetic conservation BMC Bioinformatics 2007 11 121 10.1186/1471-2105-8-121 17425808 PMC1955749

[B39] Koehntop KD Emerson JP Que L Jr The 2-His-1-carboxylate facial triad: a versatile platform for dioxygen activation by mononuclear non-heme iron(II) enzymes J Biol Inorg Chem 2005 11 2 87 93 10.1007/s00775-005-0624-x 15739104

[B40] Brenner C Hint, Fhit, and GalT: function, structure, evolution, and mechanism of three branches of the histidine triad superfamily of nucleotide hydrolases and transferases Biochemistry 2002 11 29 9003 9014 10.1021/bi025942q 12119013 PMC2571077

[B41] Brenner C Bieganowski P Pace HC Huebner K The histidine triad superfamily of nucleotide-binding proteins J Cell Physiol 1999 11 2 179 187 10.1002/(SICI)1097-4652(199911)181:2<179::AID-JCP1>3.0.CO;2-8 10497298 PMC2556047

[B42] Mashiach E Nussinov R Wolfson HJ FiberDock: Flexible induced-fit backbone refinement in molecular docking Proteins 2010 11 6 1503 1519 20077569 10.1002/prot.22668 PMC4290165

[B43] Yang JM Chen CC GEMDOCK: a generic evolutionary method for molecular docking Proteins 2004 11 2 288 304 10.1002/prot.20035 15048822

[B44] Mintz S Shulman-Peleg A Wolfson HJ Nussinov R Generation and analysis of a protein-protein interface data set with similar chemical and spatial patterns of interactions Proteins 2005 11 1 6 20 10.1002/prot.20580 16184518

[B45] Shulman-Peleg A Shatsky M Nussinov R Wolfson HJ Prediction of interacting single-stranded RNA bases by protein-binding patterns J Mol Biol 2008 11 2 299 316 10.1016/j.jmb.2008.03.043 18452949 PMC2429989

