# Supplementary material for: An interaction-motif-based scoring function for protein-ligand docking
Source: BMC Bioinformatics. 2010 Jun 2;11:298. doi: 10.1186/1471-2105-11-298 (PMC3098071; doi:10.1186/1471-2105-11-298)
Supplement: Additional file 1 — Supplementary data for details of motif related parameters. This file contains 2 tables and 3 figures. Table S1 lists the lower and upper distance thresholds for each of the 280 atom type pairs. Table S2 lists the binding site enrichment factor, Fb, of each of the 455 non-metal protein atom type triangles. Figs. S1 and S2 display the optimization results of two parameters, Penalty weight W and the raising percentage of lower thresholds, on the training dataset LPDB. Fig. S3 shows a schematic presentation for some of the atom types defined in Table 1. [file 1471-2105-11-298-S1.DOC]

**Supplementary Data**

**Table S1. The lower and upper distance thresholds for each of 280 atom type pairs.^a^**

| Interaction category^b^ | Atom type pair (Ligand-protein) | Peak | Lower threshold (Å) | Upper threshold (Å) |
| --- | --- | --- | --- | --- |
| 1 | C2P-C2P | 4.8 | 3.5 | 5.4 |
| 1 | C2P-C3P | 4.6 | 2.6 | 5.2 |
| 1 | C2P-CRP | 4.6 | 3.3 | 5.6 |
| 1 | C2P-ME | 3.0 | 2.2 | 4.0 |
| 1 | C2P-NLB | 4.0 | 3.0 | 4.4 |
| 1 | C2P-NLC | 3.8 | 1.2 | 4.4 |
| 1 | C2P-NRD | 4.0 | 3.2 | 4.6 |
| 1 | C2P-NRE | 3.8 | 2.9 | 4.4 |
| 1 | C2P-O2A | 3.8 | 3.0 | 4.5 |
| 1 | C2P-O3B | 3.8 | 1.2 | 4.5 |
| 1 | C2P-OLC | 3.8 | 2.9 | 4.2 |
| 1 | C3P-NLB | 4.0 | 3.0 | 4.4 |
| 1 | C3P-NLC | 4.2 | 3.2 | 4.6 |
| 1 | C3P-O2A | 3.8 | 2.8 | 4.2 |
| 1 | C3P-OLC | 3.6 | 2.6 | 4.5 |
| 1 | CRP-CRP | 3.8 | 3.2 | 5.4 |
| 1 | CRP-ME | 3.4 | 2.2 | 3.8 |
| 1 | CRP-NLB | 4.0 | 3.2 | 4.4 |
| 1 | CRP-OLC | 3.8 | 2.8 | 4.2 |
| 1 | HAL-CRP | 3.6 | 3.0 | 4.2 |
| 1 | HAL-ME | 3.8 | 2.8 | 4.0 |
| 1 | NLA-NLB | 3.8 | 3.2 | 4.2 |
| 1 | NLA-NLC | 3.2 | 2.8 | 4.4 |
| 1 | NLB-C2P | 4.0 | 3.0 | 4.4 |
| 1 | NLB-C3P | 3.8 | 3.1 | 4.6 |
| 1 | NLB-NLB | 4.0 | 3.0 | 4.4 |
| 1 | NLB-O2A | 3.0 | 2.4 | 4.2 |
| 1 | NLB-O3B | 3.0 | 2.2 | 4.0 |
| 1 | NLB-OLC | 3.0 | 2.1 | 4.4 |
| 1 | NLB-S3N | 4.2 | 3.2 | 4.8 |
| 1 | NLC-NLB | 4.0 | 2.6 | 4.2 |
| 1 | NLC-NLC | 4.0 | 3.2 | 4.2 |
| 1 | NLC-O2A | 3.0 | 2.7 | 3.8 |
| 1 | NLC-OLC | 3.0 | 2.2 | 3.6 |
| 1 | NRA-ME | 2.4 | 1.8 | 3.0 |
| 1 | NRA-NRD | 3.2 | 2.6 | 3.6 |
| 1 | NRA-O3B | 2.8 | 2.4 | 3.2 |
| 1 | NRA-OLC | 2.8 | 2.4 | 3.2 |
| 1 | NRD-C2P | 4.0 | 3.3 | 4.6 |
| 1 | NRD-C3P | 3.8 | 3.2 | 4.2 |
| 1 | NRD-NLB | 3.0 | 2.4 | 4.4 |
| 1 | NRD-NRD | 5.0 | 3.2 | 5.4 |
| 1 | NRD-O2A | 3.0 | 2.4 | 3.8 |
| 1 | NRD-O3B | 3.0 | 2.2 | 3.4 |
| 1 | NRD-OLC | 3.0 | 2.4 | 3.4 |
| 1 | O2A-C2P | 3.8 | 3.0 | 4.2 |
| 1 | O2A-C3P | 3.8 | 2.5 | 4.2 |
| 1 | O2A-CRP | 3.8 | 2.8 | 4.4 |
| 1 | O2A-ME | 2.2 | 1.1 | 3.4 |
| 1 | O2A-NLB | 3.0 | 2.4 | 4.4 |
| 1 | O2A-NLC | 3.0 | 1.9 | 4.4 |
| 1 | O2A-NRD | 3.0 | 2.2 | 3.8 |
| 1 | O2A-NRE | 2.8 | 2.1 | 3.2 |
| 1 | O2A-O3B | 2.8 | 1.9 | 3.2 |
| 1 | O2A-OLC | 2.8 | 1.9 | 3.2 |
| 1 | O3A-C3P | 3.6 | 3.0 | 4.2 |
| 1 | O3A-S3N | 4.0 | 3.4 | 4.2 |
| 1 | O3B-C2P | 3.8 | 2.8 | 4.2 |
| 1 | O3B-C3P | 3.8 | 2.5 | 4.4 |
| 1 | O3B-CRP | 3.6 | 2.8 | 4.2 |
| 1 | O3B-ME | 2.2 | 1.6 | 3.2 |
| 1 | O3B-NLB | 3.0 | 2.4 | 3.8 |
| 1 | O3B-NLC | 3.0 | 2.4 | 4.2 |
| 1 | O3B-NRD | 3.0 | 2.3 | 3.4 |
| 1 | O3B-NRE | 2.8 | 2.1 | 3.8 |
| 1 | O3B-O2A | 2.8 | 2.2 | 3.2 |
| 1 | O3B-O3B | 2.8 | 1.9 | 3.4 |
| 1 | O3B-OLC | 2.8 | 2.1 | 3.2 |
| 1 | P-C2P | 4.8 | 3.8 | 5.0 |
| 1 | P-C3P | 4.6 | 3.5 | 5.2 |
| 1 | P-CRP | 4.6 | 3.6 | 5.0 |
| 1 | P-ME | 3.4 | 2.6 | 4.4 |
| 1 | P-NLB | 4.0 | 3.2 | 4.4 |
| 1 | P-NLC | 4.0 | 3.1 | 4.4 |
| 1 | P-NRD | 4.2 | 3.2 | 4.6 |
| 1 | P-NRE | 3.8 | 2.9 | 4.4 |
| 1 | P-O3B | 3.8 | 2.8 | 4.2 |
| 1 | SLC-C2P | 4.8 | 4.0 | 5.2 |
| 1 | SLC-C3P | 4.4 | 2.7 | 5.2 |
| 1 | SLC-CRP | 4.6 | 3.8 | 5.2 |
| 1 | SLC-ME | 3.2 | 2.2 | 4.0 |
| 1 | SLC-NLB | 4.0 | 3.4 | 4.2 |
| 1 | SLC-NLC | 4.0 | 3.2 | 4.2 |
| 1 | SLC-NRD | 3.8 | 3.1 | 4.4 |
| 1 | SLC-NRE | 3.8 | 3.0 | 4.2 |
| 1 | SLC-O3B | 3.8 | 1.4 | 4.2 |
| 2 | C2N-C3N | N.D. | 3.2 | 4.4 |
| 2 | C2N-CRN | 5.2 | 3.4 | 5.4 |
| 2 | C2N-CRP | N.D. | 3.2 | 4.6 |
| 2 | C2N-ME | 3.6 | 2.8 | 4.2 |
| 2 | C2N-NLB | 3.8 | 3.2 | 4.2 |
| 2 | C2N-NLC | N.D. | 3.2 | 4.4 |
| 2 | C2N-NRE | N.D. | 2.2 | 4.6 |
| 2 | C2N-O3B | N.D. | 2.2 | 4.2 |
| 2 | C2N-OLC | N.D. | 2.6 | 4.4 |
| 2 | C2N-S3N | 2.0 | 1.6 | 4.2 |
| 2 | C2P-C3N | N.D. | 3.3 | 4.0^c^ |
| 2 | C2P-CRN | N.D. | 3.4 | 4.0^c^ |
| 2 | C2P-S3N | N.D. | 1.8 | 4.0^c^ |
| 2 | C3N-C3N | N.D. | 3.0 | 4.0^c^ |
| 2 | C3N-CRN | N.D. | 3.1 | 4.0^c^ |
| 2 | C3N-CRP | N.D. | 3.0 | 4.0^c^ |
| 2 | C3N-ME | N.D. | 1.4 | 4.0^c^ |
| 2 | C3N-NLB | N.D. | 3.0 | 4.0^c^ |
| 2 | C3N-NLC | N.D. | 3.0 | 4.0^c^ |
| 2 | C3N-NRE | N.D. | 2.8 | 4.0^c^ |
| 2 | C3N-O3B | N.D. | 2.5 | 4.0^c^ |
| 2 | C3N-OLC | N.D. | 2.7 | 4.0^c^ |
| 2 | C3N-S3N | N.D. | 2.6 | 4.0^c^ |
| 2 | C3P-C3N | N.D. | 3.5 | 4.0^c^ |
| 2 | C3P-C3P | 5.2 | 3.2 | 5.2 |
| 2 | C3P-CRN | N.D. | 3.2 | 5.0 |
| 2 | C3P-CRP | 4.8 | 3.3 | 5.0 |
| 2 | C3P-ME | 3.2 | 2.2 | 4.0 |
| 2 | C3P-NRE | 4.2 | 3.0 | 4.6 |
| 2 | C3P-O3B | 3.8 | 2.3 | 4.6 |
| 2 | C3P-S3N | 4.0 | 3.2 | 4.2 |
| 2 | CRN-C3N | N.D. | 3.1 | 4.0^c^ |
| 2 | CRN-CRN | 4.8 | 3.1 | 5.6 |
| 2 | CRN-CRP | N.D. | 3.2 | 4.0^c^ |
| 2 | CRN-ME | N.D. | 3.0 | 4.6 |
| 2 | CRN-NLB | 5.2 | 3.2 | 5.4 |
| 2 | CRN-NLC | N.D. | 3.0 | 4.4 |
| 2 | CRN-NRE | N.D. | 2.8 | 4.4 |
| 2 | CRN-O3B | N.D. | 2.8 | 4.0 |
| 2 | CRN-OLC | N.D. | 2.6 | 4.0 |
| 2 | CRN-S3N | N.D. | 3.2 | 4.0^c^ |
| 2 | CRP-C2P | 4.8 | 3.2 | 5.4 |
| 2 | CRP-C3N | N.D. | 3.0 | 4.0^c^ |
| 2 | CRP-C3P | N.D. | 3.2 | 5.0 |
| 2 | CRP-CRN | 4.0 | 3.1 | 4.8 |
| 2 | CRP-NLC | N.D. | 3.0 | 4.2 |
| 2 | CRP-NRE | 3.8 | 2.9 | 4.2 |
| 2 | CRP-O2A | 4.0 | 2.8 | 4.4 |
| 2 | CRP-O3B | 3.8 | 2.8 | 4.4 |
| 2 | CRP-S3N | N.D. | 3.0 | 4.4 |
| 2 | HAL-C2P | 4.4 | 2.9 | 4.6 |
| 2 | HAL-C3N | 4.0 | 3.0 | 4.2 |
| 2 | HAL-C3P | N.D. | 3.0 | 4.0^c^ |
| 2 | HAL-CRN | 4.0 | 3.0 | 5.0 |
| 2 | HAL-NLC | 3.6 | 2.4 | 4.8 |
| 2 | HAL-NRD | N.D. | 2.8 | 4.2 |
| 2 | HAL-NRE | N.D. | 2.8 | 4.2 |
| 2 | HAL-O2A | N.D. | 2.6 | 4.0^c^ |
| 2 | HAL-O3B | 3.6 | 2.4 | 4.4 |
| 2 | HAL-OLC | 3.4 | 2.8 | 3.6 |
| 2 | HAL-S3N | 3.8 | 2.8 | 4.6 |
| 2 | NLA-CRN | N.D. | 3.4 | 4.0 |
| 2 | NLA-CRP | N.D. | 3.2 | 4.0 |
| 2 | NLA-ME | 2.2 | 1.2 | 3.8 |
| 2 | NLA-NRE | N.D. | 2.6 | 4.2 |
| 2 | NLA-O3B | N.D. | 2.4 | 3.6 |
| 2 | NLA-OLC | N.D. | 2.0 | 3.2 |
| 2 | NLB-C3N | N.D. | 3.2 | 4.0^c^ |
| 2 | NLB-CRN | N.D. | 3.2 | 4.0 |
| 2 | NLB-CRP | N.D. | 3.4 | 4.2 |
| 2 | NLB-ME | 1.6 | 1.2 | 4.0 |
| 2 | NLB-NLC | N.D. | 2.5 | 4.0^c^ |
| 2 | NLB-NRD | N.D. | 2.8 | 5.4 |
| 2 | NLB-NRE | 3.8 | 2.6 | 4.0 |
| 2 | NLC-C3N | N.D. | 3.6 | 4.0^c^ |
| 2 | NLC-C3P | N.D. | 3.0 | 4.0^c^ |
| 2 | NLC-CRN | 4.8 | 3.2 | 5.4 |
| 2 | NLC-CRP | 4.4 | 3.2 | 4.8 |
| 2 | NLC-NRE | N.D. | 2.4 | 4.0^c^ |
| 2 | NLC-O3B | N.D. | 2.5 | 3.6 |
| 2 | NRA-C2P | N.D. | 3.2 | 4.4 |
| 2 | NRA-C3N | 4.8 | 3.0 | 5.0 |
| 2 | NRA-C3P | N.D. | 3.2 | 4.6 |
| 2 | NRA-CRN | 4.2 | 3.2 | 5.4 |
| 2 | NRA-CRP | 4.6 | 3.1 | 4.8 |
| 2 | NRA-NLB | N.D. | 2.8 | 4.2 |
| 2 | NRA-NLC | 4.6 | 2.8 | 5.6 |
| 2 | NRA-NRE | N.D. | 2.7 | 5.0 |
| 2 | NRA-O2A | N.D. | 2.8 | 4.4 |
| 2 | NRA-S3N | 4.6 | 3.2 | 5.0 |
| 2 | NRD-C3N | N.D. | 3.2 | 4.2 |
| 2 | NRD-CRN | N.D. | 3.2 | 4.0^c^ |
| 2 | NRD-CRP | 5.0 | 3.2 | 5.2 |
| 2 | NRD-ME | N.D. | 1.2 | 3.4 |
| 2 | NRD-NLC | N.D. | 2.8 | 4.0^c^ |
| 2 | NRD-NRE | N.D. | 2.6 | 4.0 |
| 2 | NRD-S3N | N.D. | 3.2 | 4.0^c^ |
| 2 | O2A-C3N | 3.8 | 2.6 | 4.4 |
| 2 | O2A-CRN | N.D. | 3.0 | 4.0^c^ |
| 2 | O2A-O2A | 5.0 | 2.8 | 5.6 |
| 2 | O2A-S3N | 3.4 | 2.4 | 3.8 |
| 2 | O3A-CRN | N.D. | 2.8 | 4.0^c^ |
| 2 | O3A-CRP | 5.2 | 3.1 | 5.6 |
| 2 | O3A-ME | 4.4 | 2.0 | 5.0 |
| 2 | O3A-NLB | N.D. | 2.2 | 3.6 |
| 2 | O3A-NLC | N.D. | 2.6 | 3.8 |
| 2 | O3A-NRD | N.D. | 2.7 | 4.0^c^ |
| 2 | O3A-NRE | N.D. | 2.5 | 4.0 |
| 2 | O3A-O3B | N.D. | 2.1 | 5.4 |
| 2 | O3A-OLC | N.D. | 2.6 | 5.2 |
| 2 | O3B-C3N | N.D. | 2.8 | 4.2 |
| 2 | O3B-CRN | N.D. | 2.9 | 4.0^c^ |
| 2 | O3B-S3N | N.D. | 3.0 | 3.8 |
| 2 | ORA-C3N | N.D. | 3.2 | 4.0^c^ |
| 2 | ORA-CRN | 4.0 | 3.2 | 4.6 |
| 2 | ORA-CRP | N.D. | 3.2 | 4.0 |
| 2 | ORA-NLB | N.D. | 3.0 | 4.0^c^ |
| 2 | P-C3N | N.D. | 3.4 | 4.0^c^ |
| 2 | P-O2A | N.D. | 3.4 | 4.0^c^ |
| 2 | P-OLC | N.D. | 2.8 | 4.8 |
| 2 | S3N-C3N | N.D. | 3.2 | 4.0^c^ |
| 2 | S3N-CRN | N.D. | 3.4 | 4.0^c^ |
| 2 | S3N-CRP | N.D. | 3.8 | 4.0^c^ |
| 2 | S3N-NLB | N.D. | 2.8 | 4.0^c^ |
| 2 | S3N-NLC | N.D. | 2.8 | 4.0^c^ |
| 2 | S3N-NRE | N.D. | 2.8 | 4.0^c^ |
| 2 | S3N-O3B | 3.4 | 2.8 | 4.0 |
| 2 | S3N-OLC | N.D. | 2.8 | 4.0^c^ |
| 2 | SLC-C3N | 4.8 | 3.6 | 5.0 |
| 2 | SLC-CRN | 4.6 | 3.8 | 4.6 |
| 2 | SLC-O2A | 6.0 | 3.2 | 6.4 |
| 2 | SLC-OLC | N.D. | 3.0 | 3.8 |
| 2 | SLC-S3N | N.D. | N.D. | 4.0^c^ |
| 2 | SRN-C3N | N.D. | 3.4 | 4.0^c^ |
| 2 | SRN-C3P | 4.0 | 3.5 | 4.2 |
| 2 | SRN-CRN | 4.0 | 3.4 | 4.6 |
| 2 | SRN-NRE | N.D. | 2.8 | 4.4 |
| 3 | C2N-C2P | N.D. | 3.4 | N.D. |
| 3 | C2N-C3P | N.D. | 3.2 | N.D. |
| 3 | C2N-NRD | N.D. | 3.2 | N.D. |
| 3 | C2N-O2A | N.D. | 3.0 | N.D. |
| 3 | C3N-C2P | N.D. | 3.3 | N.D. |
| 3 | C3N-C3P | N.D. | 3.4 | N.D. |
| 3 | C3N-NRD | N.D. | 3.1 | N.D. |
| 3 | C3N-O2A | N.D. | 2.8 | N.D. |
| 3 | C3P-C2P | N.D. | 3.3 | N.D. |
| 3 | C3P-NRD | N.D. | 3.1 | N.D. |
| 3 | CRN-C2P | N.D. | 3.3 | N.D. |
| 3 | CRN-C3P | N.D. | 3.0 | N.D. |
| 3 | CRN-NRD | N.D. | 3.1 | N.D. |
| 3 | CRN-O2A | N.D. | 2.9 | N.D. |
| 3 | CRP-NRD | N.D. | 3.2 | N.D. |
| 3 | NLA-C2P | N.D. | 3.4 | N.D. |
| 3 | NLA-C3N | N.D. | 3.2 | N.D. |
| 3 | NLA-C3P | N.D. | 3.2 | N.D. |
| 3 | NLA-NRD | N.D. | 2.8 | N.D. |
| 3 | NLA-O2A | N.D. | 3.0 | N.D. |
| 3 | NLC-C2P | N.D. | 3.4 | N.D. |
| 3 | NLC-NRD | N.D. | 3.3 | N.D. |
| 3 | O3A-C2P | N.D. | 3.4 | N.D. |
| 3 | O3A-C3N | N.D. | 3.0 | N.D. |
| 3 | O3A-O2A | N.D. | 3.0 | N.D. |
| 3 | ORA-C2P | N.D. | 3.8 | N.D. |
| 3 | ORA-C3P | N.D. | 3.4 | N.D. |
| 3 | ORA-NRD | N.D. | 3.0 | N.D. |
| 3 | ORA-O2A | N.D. | 3.2 | N.D. |
| 3 | P-CRN | N.D. | 3.8 | N.D. |
| 3 | P-S3N | N.D. | 3.4 | N.D. |
| 3 | S3N-C2P | N.D. | 3.6 | N.D. |
| 3 | S3N-C3P | N.D. | 3.2 | N.D. |
| 3 | S3N-NRD | N.D. | 3.0 | N.D. |
| 3 | S3N-O2A | N.D. | 3.4 | N.D. |
| 3 | SRN-C2P | N.D. | 3.4 | N.D. |
| 3 | SRN-NRD | N.D. | 3.5 | N.D. |
| 3 | SRN-O2A | N.D. | 3.2 | N.D. |
| 4 | HAL-NLB | N.D. | N.D. | N.D. |
| 4 | NLA-S3N | N.D. | N.D. | N.D. |
| 4 | NLC-ME | N.D. | N.D. | N.D. |
| 4 | NLC-S3N | N.D. | N.D. | N.D. |
| 4 | ORA-ME | N.D. | N.D. | N.D. |
| 4 | ORA-NLC | N.D. | N.D. | N.D. |
| 4 | ORA-NRE | N.D. | N.D. | N.D. |
| 4 | ORA-O3B | N.D. | N.D. | N.D. |
| 4 | ORA-OLC | N.D. | N.D. | N.D. |
| 4 | ORA-S3N | N.D. | N.D. | N.D. |
| 4 | S3N-ME | N.D. | N.D. | N.D. |
| 4 | S3N-S3N | N.D. | N.D. | N.D. |
| 4 | SRN-CRP | N.D. | N.D. | N.D. |
| 4 | SRN-ME | N.D. | N.D. | N.D. |
| 4 | SRN-NLB | N.D. | N.D. | N.D. |
| 4 | SRN-NLC | N.D. | N.D. | N.D. |
| 4 | SRN-O3B | N.D. | N.D. | N.D. |
| 4 | SRN-OLC | N.D. | N.D. | N.D. |
| 4 | SRN-S3N | N.D. | N.D. | N.D. |

^a^ N.D.=Not Determined

^b^See Fig.1

^c^Although their distributions resembled those of others in the same category on the whole, their peaks were harder to determine specifically; to simplify, their upper thresholds were set at 4Å.

**Table S2. Binding site enrichment factor, F_b_, of the 455 non-metal protein atom type triangles.**

| Rank | Triangle | F_b_ | Coverage* (Binding site) | Coverage* (whole protein) |
| --- | --- | --- | --- | --- |
| 1 | NLC-NLC-NLC | 70.6 | 13.4 | 93.7 |
| 2 | CRP-CRP-CRP | 61.9 | 23.7 | 96.6 |
| 3 | CRP-NLC-NLC | 47.2 | 16.8 | 98.0 |
| 4 | CRP-CRP-NLC | 44.3 | 20.4 | 97.7 |
| 5 | CRP-CRP-OLC | 38.1 | 23.0 | 98.3 |
| 6 | NLC-NLC-NRE | 32.8 | 8.4 | 95.7 |
| 7 | CRP-CRP-NRE | 31.7 | 17.0 | 93.6 |
| 8 | CRP-CRP-O3B | 29.8 | 26.9 | 98.2 |
| 9 | NLC-NLC-O3B | 29.6 | 16.5 | 98.6 |
| 10 | CRP-CRP-NLB | 26.2 | 20.1 | 98.1 |
| 11 | CRN-CRP-CRP | 26.2 | 32.9 | 98.5 |
| 12 | CRP-OLC-OLC | 25.8 | 21.4 | 98.8 |
| 13 | NLB-NLC-NLC | 24.6 | 20.7 | 98.9 |
| 14 | CRP-NRE-OLC | 23.5 | 15.7 | 97.6 |
| 15 | CRP-NLC-O3B | 23.5 | 19.4 | 98.3 |
| 16 | CRP-NLC-NRE | 23.0 | 12.3 | 96.9 |
| 17 | NRE-OLC-OLC | 19.9 | 12.7 | 97.9 |
| 18 | CRP-NRE-NRE | 19.7 | 9.2 | 89.4 |
| 19 | CRP-NLB-NLC | 19.2 | 17.5 | 98.3 |
| 20 | NLC-O3B-O3B | 18.6 | 14.5 | 98.5 |
| 21 | NRE-NRE-OLC | 18.1 | 8.4 | 96.7 |
| 22 | CRN-NLC-NLC | 17.5 | 17.6 | 98.6 |
| 23 | NRE-NRE-NRE | 17.3 | 4.3 | 77.3 |
| 24 | OLC-OLC-OLC | 17.0 | 13.2 | 98.4 |
| 25 | CRP-NLC-OLC | 17.0 | 16.1 | 98.3 |
| 26 | CRN-CRN-CRN | 16.9 | 42.1 | 99.0 |
| 27 | CRP-CRP-S3N | 16.8 | 5.3 | 93.6 |
| 28 | NLC-NRE-NRE | 16.7 | 6.2 | 94.2 |
| 29 | CRN-CRN-CRP | 16.5 | 38.2 | 98.9 |
| 30 | C3P-NLC-NLC | 16.2 | 34.1 | 98.9 |
| 31 | CRP-O3B-O3B | 16.1 | 16.0 | 98.1 |
| 32 | CRN-CRP-NRE | 16.1 | 20.7 | 97.6 |
| 33 | CRN-CRP-OLC | 15.8 | 24.7 | 98.8 |
| 34 | CRN-CRN-NRE | 15.8 | 22.6 | 97.9 |
| 35 | NLC-NLC-S3N | 15.7 | 3.1 | 93.2 |
| 36 | CRN-CRP-NLC | 15.6 | 22.2 | 98.6 |
| 37 | C3P-CRP-CRP | 15.0 | 45.6 | 98.6 |
| 38 | CRP-NRE-O3B | 14.8 | 15.9 | 97.0 |
| 39 | CRP-NLB-NRE | 14.0 | 12.3 | 97.3 |
| 40 | O3B-O3B-O3B | 13.8 | 9.4 | 98.0 |
| 41 | NLB-NLC-NRE | 13.8 | 9.9 | 96.8 |
| 42 | CRP-O3B-OLC | 13.7 | 19.1 | 98.8 |
| 43 | NLC-NRE-O3B | 13.7 | 9.6 | 97.1 |
| 44 | CRN-NRE-NRE | 13.4 | 11.0 | 96.4 |
| 45 | NLC-NLC-OLC | 13.3 | 14.7 | 98.8 |
| 46 | CRN-NRE-OLC | 12.1 | 14.5 | 97.8 |
| 47 | CRN-CRP-O3B | 12.1 | 30.2 | 98.8 |
| 48 | CRN-NLC-NRE | 11.7 | 12.1 | 97.3 |
| 49 | CRP-NLB-OLC | 11.7 | 14.5 | 98.6 |
| 50 | CRP-NLC-S3N | 11.2 | 3.8 | 93.8 |
| 51 | NLC-NRE-OLC | 11.1 | 8.8 | 97.2 |
| 52 | CRP-NLB-O3B | 10.8 | 16.1 | 98.6 |
| 53 | C3P-CRP-NLC | 10.8 | 31.7 | 98.7 |
| 54 | O3B-OLC-OLC | 10.8 | 23.0 | 98.9 |
| 55 | CRN-OLC-OLC | 10.7 | 22.3 | 98.9 |
| 56 | CRP-NRE-S3N | 10.7 | 2.8 | 91.4 |
| 57 | NLB-NRE-NRE | 10.6 | 5.8 | 94.3 |
| 58 | CRN-CRN-NLC | 10.4 | 24.5 | 98.8 |
| 59 | CRP-S3N-S3N | 10.4 | 1.1 | 83.0 |
| 60 | NRE-NRE-O3B | 10.2 | 7.2 | 94.5 |
| 61 | CRN-CRP-NLB | 10.0 | 20.6 | 98.8 |
| 62 | NLB-NLC-O3B | 9.8 | 17.9 | 98.9 |
| 63 | NLC-NLC-O2A | 9.8 | 27.0 | 98.9 |
| 64 | NRE-S3N-S3N | 9.5 | 0.7 | 75.4 |
| 65 | CRN-NLC-O3B | 9.4 | 22.0 | 98.8 |
| 66 | CRN-NLB-NLC | 9.4 | 18.3 | 98.7 |
| 67 | NLB-NLB-NLC | 9.3 | 9.1 | 98.6 |
| 68 | OLC-OLC-S3N | 9.3 | 7.6 | 95.9 |
| 69 | CRN-CRN-O3B | 9.2 | 35.0 | 98.9 |
| 70 | C3P-NLC-O3B | 9.1 | 35.0 | 99.0 |
| 71 | CRN-CRN-OLC | 9.0 | 25.6 | 98.9 |
| 72 | NLC-OLC-OLC | 8.7 | 18.1 | 98.9 |
| 73 | CRN-CRN-NLB | 8.6 | 22.1 | 98.9 |
| 74 | CRP-NLB-NLB | 8.3 | 6.5 | 98.1 |
| 75 | O3B-O3B-OLC | 8.3 | 11.4 | 98.8 |
| 76 | NRE-NRE-S3N | 8.2 | 1.1 | 83.7 |
| 77 | C3N-NLC-NLC | 8.2 | 29.0 | 98.9 |
| 78 | NRE-O3B-OLC | 8.1 | 10.0 | 97.8 |
| 79 | NLB-NLC-S3N | 8.1 | 4.1 | 95.0 |
| 80 | CRN-NLB-NRE | 8.1 | 11.3 | 97.7 |
| 81 | C3P-CRP-NRE | 8.0 | 29.8 | 97.9 |
| 82 | NLC-O3B-OLC | 8.0 | 15.8 | 98.9 |
| 83 | CRP-NLB-S3N | 7.9 | 3.2 | 94.6 |
| 84 | NLB-NRE-OLC | 7.8 | 8.1 | 97.4 |
| 85 | C3P-CRP-O3B | 7.7 | 40.6 | 98.8 |
| 86 | CRN-NRE-O3B | 7.6 | 17.2 | 97.9 |
| 87 | NRE-O3B-O3B | 7.5 | 7.0 | 96.8 |
| 88 | CRP-OLC-S3N | 7.4 | 4.0 | 95.1 |
| 89 | CRP-CRP-O2A | 7.3 | 36.3 | 98.6 |
| 90 | CRP-O3B-S3N | 7.3 | 5.0 | 95.3 |
| 91 | CRN-O3B-O3B | 7.2 | 20.1 | 98.8 |
| 92 | C3P-O3B-O3B | 7.2 | 30.1 | 98.9 |
| 93 | C3P-NLC-NRE | 7.1 | 18.2 | 97.7 |
| 94 | CRN-NLC-OLC | 7.1 | 18.1 | 98.8 |
| 95 | NLC-NRE-S3N | 7.0 | 1.6 | 89.4 |
| 96 | CRN-O3B-OLC | 6.8 | 22.5 | 98.9 |
| 97 | NLB-OLC-OLC | 6.8 | 17.2 | 98.9 |
| 98 | C3P-CRP-OLC | 6.8 | 34.2 | 98.9 |
| 99 | NLB-O3B-O3B | 6.6 | 9.5 | 98.8 |
| 100 | C2P-CRP-CRP | 6.6 | 30.7 | 98.6 |
| 101 | CRN-CRP-S3N | 6.5 | 7.0 | 95.6 |
| 102 | C3N-CRP-CRP | 6.2 | 39.0 | 98.6 |
| 103 | C2P-NLC-NLC | 6.2 | 20.4 | 98.9 |
| 104 | C3P-NLB-NLC | 6.2 | 33.3 | 99.0 |
| 105 | NLC-O3B-S3N | 6.0 | 3.2 | 95.3 |
| 106 | NLB-NRE-S3N | 5.9 | 1.8 | 90.9 |
| 107 | C3P-NRE-NRE | 5.9 | 15.6 | 97.2 |
| 108 | CRN-NRE-S3N | 5.8 | 4.0 | 93.1 |
| 109 | NLB-NLC-OLC | 5.8 | 15.6 | 98.9 |
| 110 | CRN-NLB-OLC | 5.7 | 14.1 | 98.8 |
| 111 | C3P-OLC-OLC | 5.7 | 43.4 | 99.0 |
| 112 | C3P-C3P-NLC | 5.6 | 48.7 | 99.0 |
| 113 | C3P-CRP-NLB | 5.5 | 30.4 | 98.8 |
| 114 | NLB-NRE-O3B | 5.5 | 8.1 | 97.5 |
| 115 | NLB-NLB-NRE | 5.5 | 3.3 | 95.7 |
| 116 | CRN-NLC-S3N | 5.4 | 4.6 | 95.5 |
| 117 | CRN-CRN-S3N | 5.3 | 9.5 | 96.0 |
| 118 | NRE-OLC-S3N | 5.1 | 1.9 | 91.9 |
| 119 | CRN-OLC-S3N | 5.0 | 5.8 | 95.9 |
| 120 | NRE-O3B-S3N | 5.0 | 2.5 | 92.8 |
| 121 | CRP-NLC-O2A | 5.0 | 24.3 | 98.7 |
| 122 | NLC-NLC-NRD | 4.9 | 16.3 | 98.9 |
| 123 | C3P-CRN-CRP | 4.9 | 47.2 | 98.9 |
| 124 | O3B-OLC-S3N | 4.8 | 5.5 | 96.0 |
| 125 | NLC-OLC-S3N | 4.8 | 3.2 | 95.3 |
| 126 | CRN-NLB-O3B | 4.7 | 18.8 | 98.9 |
| 127 | CRN-NLB-NLB | 4.6 | 7.1 | 98.6 |
| 128 | C3N-CRP-NLC | 4.5 | 28.3 | 98.7 |
| 129 | C3P-C3P-CRP | 4.5 | 56.5 | 98.9 |
| 130 | CRP-CRP-NRD | 4.4 | 25.7 | 98.6 |
| 131 | C3P-NRE-OLC | 4.4 | 20.0 | 98.1 |
| 132 | NLB-NLC-O2A | 4.4 | 29.4 | 99.0 |
| 133 | C3P-O3B-OLC | 4.3 | 37.0 | 98.9 |
| 134 | C2P-NLC-O3B | 4.3 | 23.9 | 98.9 |
| 135 | C3P-C3P-O3B | 4.3 | 58.9 | 99.0 |
| 136 | NLB-O3B-OLC | 4.3 | 13.5 | 98.9 |
| 137 | C3N-NLB-NLC | 4.2 | 32.1 | 99.0 |
| 138 | C3P-CRP-S3N | 4.2 | 10.6 | 96.0 |
| 139 | CRN-NLB-S3N | 4.1 | 4.5 | 95.9 |
| 140 | C3P-CRN-NLC | 4.1 | 34.1 | 98.9 |
| 141 | C3P-NRE-O3B | 4.0 | 24.4 | 98.2 |
| 142 | C2P-O3B-O3B | 4.0 | 22.6 | 98.9 |
| 143 | NLC-O2A-O3B | 4.0 | 26.3 | 99.0 |
| 144 | C3P-NLC-S3N | 3.9 | 8.3 | 96.1 |
| 145 | NLC-NRD-O3B | 3.9 | 21.7 | 99.0 |
| 146 | C3P-NLC-OLC | 3.9 | 30.8 | 98.9 |
| 147 | C2P-CRP-NLC | 3.8 | 19.4 | 98.7 |
| 148 | CRP-NLB-O2A | 3.8 | 25.1 | 98.8 |
| 149 | NLB-NLB-OLC | 3.7 | 5.4 | 98.7 |
| 150 | CRN-S3N-S3N | 3.7 | 1.5 | 86.5 |
| 151 | NLB-NLB-S3N | 3.7 | 1.4 | 93.2 |
| 152 | OLC-S3N-S3N | 3.7 | 0.8 | 82.8 |
| 153 | C2P-CRP-O3B | 3.7 | 28.3 | 98.8 |
| 154 | NLB-NLB-O3B | 3.6 | 5.2 | 98.8 |
| 155 | NRD-O3B-O3B | 3.6 | 19.7 | 98.9 |
| 156 | C3P-NLB-O3B | 3.6 | 31.5 | 98.9 |
| 157 | O3B-O3B-S3N | 3.5 | 2.9 | 95.3 |
| 158 | CRP-NRE-O2A | 3.5 | 20.8 | 97.9 |
| 159 | C3N-CRP-NRE | 3.5 | 23.8 | 97.9 |
| 160 | NLC-NRE-O2A | 3.4 | 12.7 | 97.7 |
| 161 | C3P-CRN-NRE | 3.4 | 28.7 | 98.2 |
| 162 | C3P-NLB-NRE | 3.4 | 18.1 | 98.1 |
| 163 | S3N-S3N-S3N | 3.4 | 0.3 | 57.3 |
| 164 | NLB-S3N-S3N | 3.4 | 0.6 | 83.5 |
| 165 | NLB-OLC-S3N | 3.4 | 2.8 | 95.9 |
| 166 | NRD-OLC-S3N | 3.3 | 7.8 | 96.4 |
| 167 | CRP-O2A-O3B | 3.3 | 30.9 | 98.8 |
| 168 | C3P-OLC-S3N | 3.2 | 12.0 | 96.4 |
| 169 | C3N-NLC-NRE | 3.2 | 14.5 | 97.7 |
| 170 | C3P-C3P-C3P | 3.1 | 74.9 | 99.5 |
| 171 | C3P-NLC-O2A | 3.1 | 46.9 | 99.0 |
| 172 | C3N-NLC-O3B | 3.0 | 30.1 | 99.0 |
| 173 | C3P-NRE-S3N | 3.0 | 6.3 | 94.3 |
| 174 | C2P-CRP-NRE | 3.0 | 17.0 | 97.9 |
| 175 | C3N-NRE-NRE | 3.0 | 12.5 | 97.2 |
| 176 | NLC-S3N-S3N | 3.0 | 0.5 | 79.2 |
| 177 | C3P-CRN-CRN | 3.0 | 51.3 | 99.0 |
| 178 | NLB-O3B-S3N | 3.0 | 2.7 | 96.0 |
| 179 | O2A-O3B-O3B | 2.9 | 21.3 | 98.9 |
| 180 | CRN-O3B-S3N | 2.9 | 6.4 | 96.0 |
| 181 | C3P-C3P-OLC | 2.9 | 52.3 | 99.0 |
| 182 | C3P-CRN-O3B | 2.8 | 46.0 | 98.9 |
| 183 | C3P-CRN-OLC | 2.8 | 35.9 | 98.9 |
| 184 | CRP-NRD-O3B | 2.8 | 25.6 | 98.8 |
| 185 | C3N-CRP-O3B | 2.7 | 33.0 | 98.8 |
| 186 | C2P-C3P-NLC | 2.7 | 41.4 | 99.0 |
| 187 | CRP-NLC-NRD | 2.7 | 16.5 | 98.7 |
| 188 | C3N-CRP-NLB | 2.7 | 26.5 | 98.8 |
| 189 | C2P-CRP-S3N | 2.7 | 6.5 | 96.0 |
| 190 | C3P-C3P-NRE | 2.7 | 35.2 | 98.3 |
| 191 | C3N-CRN-CRP | 2.6 | 43.5 | 98.9 |
| 192 | C2P-NLB-NLC | 2.6 | 22.0 | 99.0 |
| 193 | C3N-CRN-NLC | 2.6 | 31.6 | 98.9 |
| 194 | C3N-CRN-CRN | 2.6 | 50.5 | 99.0 |
| 195 | C3P-NLB-OLC | 2.5 | 26.4 | 99.0 |
| 196 | C3P-C3P-NLB | 2.5 | 43.7 | 99.2 |
| 197 | NRE-NRE-O2A | 2.5 | 10.1 | 97.2 |
| 198 | C3N-CRP-OLC | 2.5 | 28.2 | 98.9 |
| 199 | C2P-C3P-O3B | 2.5 | 52.3 | 99.0 |
| 200 | NLC-O2A-O2A | 2.5 | 35.0 | 99.0 |
| 201 | CRN-CRP-O2A | 2.4 | 38.8 | 98.9 |
| 202 | NLB-NLB-NLB | 2.4 | 1.3 | 96.2 |
| 203 | CRP-O2A-OLC | 2.4 | 24.7 | 98.9 |
| 204 | NRD-OLC-OLC | 2.4 | 26.4 | 99.0 |
| 205 | C2P-CRP-OLC | 2.4 | 21.9 | 98.9 |
| 206 | C2P-C3P-CRP | 2.4 | 48.6 | 98.9 |
| 207 | C3P-NLB-NLB | 2.4 | 13.5 | 98.9 |
| 208 | C2P-CRP-NLB | 2.3 | 18.5 | 98.8 |
| 209 | C3P-CRN-NLB | 2.3 | 31.0 | 99.0 |
| 210 | NLB-NRE-O2A | 2.3 | 14.7 | 98.1 |
| 211 | C3P-CRP-O2A | 2.3 | 53.7 | 98.9 |
| 212 | C3P-NLB-S3N | 2.3 | 8.3 | 96.4 |
| 213 | C2P-OLC-OLC | 2.3 | 28.9 | 99.0 |
| 214 | NLB-NLB-O2A | 2.3 | 12.7 | 98.9 |
| 215 | C3P-NLC-NRD | 2.2 | 37.1 | 99.0 |
| 216 | C2P-O3B-OLC | 2.2 | 25.3 | 98.9 |
| 217 | C3N-C3P-NLC | 2.2 | 51.1 | 99.0 |
| 218 | CRN-NLC-O2A | 2.2 | 27.9 | 98.9 |
| 219 | NRD-O3B-OLC | 2.2 | 24.0 | 98.9 |
| 220 | O3B-S3N-S3N | 2.2 | 0.8 | 86.1 |
| 221 | C2P-CRN-CRP | 2.2 | 32.0 | 98.9 |
| 222 | C2P-NLC-NRE | 2.2 | 9.2 | 97.7 |
| 223 | C3N-NLC-S3N | 2.2 | 7.3 | 96.1 |
| 224 | C3P-O3B-S3N | 2.2 | 12.4 | 96.4 |
| 225 | C3N-CRN-NRE | 2.1 | 25.3 | 98.2 |
| 226 | CRP-O2A-S3N | 2.1 | 7.2 | 96.0 |
| 227 | CRP-NRD-NRE | 2.1 | 14.4 | 97.9 |
| 228 | C3P-NRD-O3B | 2.1 | 49.1 | 99.0 |
| 229 | NLB-O2A-O3B | 2.1 | 24.7 | 98.9 |
| 230 | C3N-CRP-S3N | 2.0 | 8.6 | 96.0 |
| 231 | C2P-OLC-S3N | 2.0 | 8.5 | 96.4 |
| 232 | NLC-O2A-S3N | 2.0 | 6.5 | 96.1 |
| 233 | C2P-NRE-NRE | 2.0 | 7.5 | 97.2 |
| 234 | C2P-NRE-S3N | 2.0 | 3.2 | 94.3 |
| 235 | C3N-OLC-OLC | 2.0 | 35.1 | 99.0 |
| 236 | NLB-O2A-S3N | 2.0 | 7.6 | 96.4 |
| 237 | O2A-OLC-OLC | 2.0 | 31.3 | 99.0 |
| 238 | C3P-O2A-O3B | 1.9 | 56.6 | 99.0 |
| 239 | NLB-NLC-NRD | 1.9 | 19.6 | 99.0 |
| 240 | C2P-C3P-C3P | 1.9 | 70.6 | 99.2 |
| 241 | CRP-NRD-OLC | 1.9 | 19.5 | 98.9 |
| 242 | CRN-CRN-O2A | 1.9 | 43.2 | 99.0 |
| 243 | C3N-NLB-NRE | 1.9 | 15.2 | 98.1 |
| 244 | C2P-NLC-S3N | 1.9 | 5.6 | 96.1 |
| 245 | C3N-O3B-O3B | 1.9 | 22.5 | 98.9 |
| 246 | C3P-C3P-S3N | 1.9 | 19.2 | 96.5 |
| 247 | C2P-NLB-O3B | 1.8 | 19.8 | 98.9 |
| 248 | C3N-NLC-OLC | 1.8 | 27.1 | 98.9 |
| 249 | C3N-NRE-S3N | 1.8 | 4.3 | 94.3 |
| 250 | CRP-O2A-O2A | 1.8 | 37.0 | 98.9 |
| 251 | C3P-S3N-S3N | 1.8 | 2.3 | 88.3 |
| 252 | CRN-NLB-O2A | 1.8 | 26.7 | 98.9 |
| 253 | C3P-NLB-O2A | 1.7 | 46.2 | 99.1 |
| 254 | O2A-OLC-S3N | 1.7 | 8.6 | 96.4 |
| 255 | C3P-C3P-O2A | 1.7 | 77.4 | 99.2 |
| 256 | C3N-NRE-OLC | 1.7 | 16.0 | 98.1 |
| 257 | C3P-CRN-S3N | 1.7 | 12.3 | 96.3 |
| 258 | NRD-NRE-NRE | 1.7 | 6.5 | 97.2 |
| 259 | C2P-CRN-NLC | 1.7 | 21.2 | 98.9 |
| 260 | NLB-O2A-O2A | 1.7 | 36.0 | 99.1 |
| 261 | NRE-O2A-S3N | 1.7 | 3.6 | 94.3 |
| 262 | CRP-NLB-NRD | 1.7 | 15.6 | 98.8 |
| 263 | C3N-CRN-NLB | 1.6 | 28.8 | 99.0 |
| 264 | C2P-NRE-O3B | 1.6 | 12.8 | 98.1 |
| 265 | CRN-NRE-O2A | 1.6 | 21.3 | 98.2 |
| 266 | C2P-C2P-O3B | 1.6 | 37.5 | 99.0 |
| 267 | C3N-NLB-NLB | 1.6 | 11.9 | 98.9 |
| 268 | C2P-C2P-CRP | 1.6 | 33.1 | 98.9 |
| 269 | CRP-NRD-S3N | 1.6 | 6.0 | 96.0 |
| 270 | NRE-O2A-O3B | 1.6 | 15.9 | 98.2 |
| 271 | C3P-C3P-CRN | 1.6 | 56.9 | 99.0 |
| 272 | C3P-CRP-NRD | 1.6 | 43.0 | 98.9 |
| 273 | NLC-NRD-NRE | 1.6 | 7.3 | 97.7 |
| 274 | C2P-C3P-OLC | 1.5 | 46.1 | 99.0 |
| 275 | C3N-NLC-O2A | 1.5 | 44.6 | 99.0 |
| 276 | C2P-CRN-CRN | 1.5 | 35.1 | 99.0 |
| 277 | NRD-NRD-S3N | 1.5 | 10.1 | 96.5 |
| 278 | O2A-O3B-OLC | 1.5 | 25.7 | 98.9 |
| 279 | NLB-NRD-O3B | 1.5 | 17.5 | 98.9 |
| 280 | C3N-NRE-O3B | 1.5 | 17.3 | 98.2 |
| 281 | NLC-O2A-OLC | 1.5 | 22.0 | 98.9 |
| 282 | C3P-C3P-NRD | 1.5 | 65.3 | 99.2 |
| 283 | NLC-NRD-S3N | 1.5 | 4.5 | 96.1 |
| 284 | C3N-C3P-CRP | 1.5 | 56.5 | 98.9 |
| 285 | NRD-NRD-O3B | 1.5 | 33.4 | 99.0 |
| 286 | NRD-NRD-OLC | 1.5 | 26.3 | 99.0 |
| 287 | C3N-CRN-OLC | 1.4 | 31.8 | 98.9 |
| 288 | NRE-O2A-OLC | 1.4 | 12.3 | 98.1 |
| 289 | NLC-NRD-O2A | 1.4 | 33.3 | 99.0 |
| 290 | C2P-C3P-NLB | 1.4 | 38.1 | 99.1 |
| 291 | C2P-NLB-S3N | 1.4 | 5.8 | 96.4 |
| 292 | NRD-NRE-O3B | 1.4 | 12.1 | 98.2 |
| 293 | C2P-C2P-NLC | 1.4 | 26.7 | 99.0 |
| 294 | C2P-NLC-O2A | 1.4 | 34.2 | 99.0 |
| 295 | C3N-NLB-O3B | 1.4 | 25.6 | 98.9 |
| 296 | C3N-NLB-S3N | 1.4 | 7.4 | 96.4 |
| 297 | C3N-S3N-S3N | 1.4 | 2.2 | 88.3 |
| 298 | C3N-C3N-NLC | 1.4 | 41.0 | 99.0 |
| 299 | C3N-CRN-O3B | 1.4 | 41.7 | 98.9 |
| 300 | C3P-NRD-OLC | 1.4 | 42.5 | 99.0 |
| 301 | C3N-CRN-S3N | 1.4 | 11.7 | 96.3 |
| 302 | NLB-O2A-OLC | 1.4 | 21.7 | 99.0 |
| 303 | C3P-NRE-O2A | 1.4 | 32.6 | 98.3 |
| 304 | C2P-CRN-O3B | 1.3 | 30.7 | 98.9 |
| 305 | CRN-O2A-O3B | 1.3 | 35.7 | 98.9 |
| 306 | C2P-NLB-NRE | 1.3 | 9.7 | 98.1 |
| 307 | C3P-O2A-O2A | 1.3 | 69.7 | 99.2 |
| 308 | C2P-CRN-NRE | 1.3 | 15.7 | 98.2 |
| 309 | O2A-O2A-O3B | 1.3 | 39.0 | 99.0 |
| 310 | CRN-CRP-NRD | 1.3 | 28.0 | 98.9 |
| 311 | C2P-C2P-C3P | 1.3 | 61.8 | 99.2 |
| 312 | NLB-NRD-S3N | 1.3 | 5.3 | 96.4 |
| 313 | C3N-O3B-OLC | 1.3 | 28.2 | 98.9 |
| 314 | C2P-C3P-NRE | 1.3 | 28.1 | 98.3 |
| 315 | C2P-NRD-O3B | 1.2 | 39.2 | 99.0 |
| 316 | C3P-O2A-OLC | 1.2 | 49.8 | 99.0 |
| 317 | C2P-NLC-OLC | 1.2 | 18.0 | 98.9 |
| 318 | C2P-C3P-S3N | 1.2 | 17.9 | 96.5 |
| 319 | C3N-OLC-S3N | 1.2 | 8.3 | 96.4 |
| 320 | C3N-NLB-OLC | 1.2 | 22.6 | 99.0 |
| 321 | C3P-NRD-S3N | 1.2 | 16.9 | 96.5 |
| 322 | O2A-O2A-O2A | 1.2 | 51.7 | 99.2 |
| 323 | C2P-S3N-S3N | 1.2 | 1.5 | 88.3 |
| 324 | NRD-O3B-S3N | 1.2 | 7.5 | 96.4 |
| 325 | C2P-NRE-OLC | 1.2 | 9.7 | 98.1 |
| 326 | C2P-O3B-S3N | 1.2 | 7.8 | 96.4 |
| 327 | C2P-CRP-O2A | 1.2 | 38.0 | 98.9 |
| 328 | C3P-O2A-S3N | 1.2 | 19.4 | 96.5 |
| 329 | NRD-O2A-O3B | 1.2 | 43.4 | 99.0 |
| 330 | C2P-NLC-NRD | 1.1 | 26.7 | 99.0 |
| 331 | NRD-NRE-S3N | 1.1 | 2.9 | 94.3 |
| 332 | NLC-NRD-NRD | 1.1 | 20.4 | 99.0 |
| 333 | C2P-NLB-NLB | 1.1 | 8.0 | 98.9 |
| 334 | NRD-NRD-NRD | 1.1 | 34.9 | 99.2 |
| 335 | O2A-S3N-S3N | 1.1 | 1.9 | 88.3 |
| 336 | NRD-NRE-OLC | 1.1 | 8.6 | 98.1 |
| 337 | C3N-C3P-NLB | 1.1 | 47.2 | 99.1 |
| 338 | C2P-CRN-S3N | 1.1 | 8.3 | 96.3 |
| 339 | C2P-CRN-OLC | 1.1 | 21.6 | 98.9 |
| 340 | CRP-NRD-O2A | 1.1 | 37.3 | 98.9 |
| 341 | C2P-NLB-OLC | 1.1 | 16.6 | 99.0 |
| 342 | NRE-O2A-O2A | 1.1 | 20.1 | 98.3 |
| 343 | NLC-NRD-OLC | 1.1 | 15.9 | 98.9 |
| 344 | C3N-C3P-O3B | 1.1 | 58.3 | 99.0 |
| 345 | C2P-C3N-NLC | 1.1 | 37.4 | 99.0 |
| 346 | C2P-O2A-O3B | 1.0 | 42.7 | 99.0 |
| 347 | O2A-O3B-S3N | 1.0 | 8.7 | 96.4 |
| 348 | C3P-NRD-NRD | 1.0 | 54.0 | 99.2 |
| 349 | CRN-O2A-S3N | 1.0 | 9.6 | 96.3 |
| 350 | C2P-CRN-NLB | 1.0 | 18.2 | 98.9 |
| 351 | C3N-NLB-O2A | 1.0 | 44.4 | 99.1 |
| 352 | C2P-C2P-OLC | 1.0 | 30.0 | 99.0 |
| 353 | C3P-NLB-NRD | 1.0 | 34.9 | 99.1 |
| 354 | CRN-O2A-OLC | 1.0 | 25.4 | 98.9 |
| 355 | CRN-NLC-NRD | 1.0 | 16.1 | 98.9 |
| 356 | C3N-CRP-O2A | 1.0 | 48.2 | 98.9 |
| 357 | C2P-C3P-NRD | 1.0 | 63.0 | 99.2 |
| 358 | C3P-NRD-O2A | 1.0 | 68.7 | 99.2 |
| 359 | C2P-C3P-O2A | 1.0 | 69.9 | 99.2 |
| 360 | C3N-C3P-NRE | 1.0 | 34.3 | 98.3 |
| 361 | NLB-NRD-NRE | 0.9 | 7.5 | 98.1 |
| 362 | C2P-CRP-NRD | 0.9 | 32.9 | 98.9 |
| 363 | NLB-NLB-NRD | 0.9 | 6.7 | 98.9 |
| 364 | C2P-C2P-C2P | 0.9 | 42.4 | 99.2 |
| 365 | CRN-CRN-NRD | 0.9 | 28.8 | 99.0 |
| 366 | NRD-O2A-S3N | 0.9 | 15.5 | 96.5 |
| 367 | C2P-C2P-NLB | 0.9 | 23.3 | 99.1 |
| 368 | C3P-CRN-O2A | 0.9 | 56.7 | 99.0 |
| 369 | NLB-NRD-OLC | 0.9 | 14.4 | 99.0 |
| 370 | C2P-NLB-O2A | 0.9 | 33.0 | 99.1 |
| 371 | C2P-C2P-S3N | 0.9 | 11.8 | 96.5 |
| 372 | CRN-NRD-O3B | 0.9 | 27.1 | 98.9 |
| 373 | C3N-C3P-C3P | 0.9 | 78.8 | 99.2 |
| 374 | C3N-C3P-OLC | 0.9 | 52.7 | 99.0 |
| 375 | NLB-NRD-O2A | 0.9 | 32.7 | 99.1 |
| 376 | C2P-C3P-CRN | 0.9 | 48.8 | 99.0 |
| 377 | NRD-O2A-O2A | 0.9 | 58.8 | 99.2 |
| 378 | CRP-NRD-NRD | 0.9 | 24.8 | 98.9 |
| 379 | C2P-NRD-OLC | 0.9 | 31.9 | 99.0 |
| 380 | O2A-O2A-S3N | 0.9 | 14.2 | 96.5 |
| 381 | C3P-NRD-NRE | 0.9 | 24.4 | 98.3 |
| 382 | NRD-NRD-O2A | 0.9 | 52.1 | 99.2 |
| 383 | C3N-NLC-NRD | 0.8 | 33.9 | 99.0 |
| 384 | CRN-NRD-OLC | 0.8 | 19.3 | 98.9 |
| 385 | NRD-O2A-OLC | 0.8 | 35.9 | 99.0 |
| 386 | C2P-NRD-S3N | 0.8 | 12.9 | 96.5 |
| 387 | C2P-C3N-CRP | 0.8 | 42.3 | 98.9 |
| 388 | CRN-NRD-NRE | 0.8 | 13.3 | 98.2 |
| 389 | C3N-O3B-S3N | 0.8 | 7.9 | 96.4 |
| 390 | C3N-C3N-CRP | 0.8 | 42.6 | 98.9 |
| 391 | C3N-C3P-CRN | 0.7 | 60.0 | 99.0 |
| 392 | CRN-NLB-NRD | 0.7 | 15.3 | 99.0 |
| 393 | C2P-C2P-NRE | 0.7 | 14.1 | 98.3 |
| 394 | CRN-O2A-O2A | 0.7 | 40.2 | 99.0 |
| 395 | C3N-C3N-NLB | 0.7 | 36.2 | 99.1 |
| 396 | O2A-O2A-OLC | 0.7 | 29.7 | 99.0 |
| 397 | C3N-C3P-S3N | 0.7 | 19.1 | 96.5 |
| 398 | C2P-O2A-S3N | 0.7 | 15.0 | 96.5 |
| 399 | CRN-NRD-S3N | 0.7 | 7.5 | 96.3 |
| 400 | C2P-NRD-NRD | 0.7 | 41.8 | 99.2 |
| 401 | NRD-S3N-S3N | 0.7 | 1.3 | 88.3 |
| 402 | C3N-NRE-O2A | 0.7 | 28.1 | 98.3 |
| 403 | C3N-C3N-CRN | 0.7 | 50.5 | 99.0 |
| 404 | C2P-O2A-O2A | 0.7 | 54.2 | 99.2 |
| 405 | C3N-C3P-O2A | 0.6 | 79.3 | 99.2 |
| 406 | C2P-C3N-NLB | 0.6 | 34.3 | 99.1 |
| 407 | C3N-O2A-O3B | 0.6 | 49.1 | 99.0 |
| 408 | C3N-C3N-NRE | 0.6 | 22.6 | 98.3 |
| 409 | C2P-C2P-NRD | 0.6 | 44.9 | 99.2 |
| 410 | NRD-NRE-O2A | 0.6 | 20.3 | 98.3 |
| 411 | C2P-NLB-NRD | 0.6 | 25.0 | 99.1 |
| 412 | C2P-C2P-CRN | 0.6 | 32.0 | 99.0 |
| 413 | C3N-O2A-O2A | 0.6 | 65.2 | 99.2 |
| 414 | C3N-CRP-NRD | 0.6 | 37.0 | 98.9 |
| 415 | C2P-NRE-O2A | 0.6 | 19.8 | 98.3 |
| 416 | C2P-O2A-OLC | 0.6 | 34.2 | 99.0 |
| 417 | C2P-C3N-O3B | 0.6 | 45.3 | 99.0 |
| 418 | C3N-CRN-O2A | 0.6 | 53.3 | 99.0 |
| 419 | C2P-C2P-O2A | 0.6 | 50.4 | 99.2 |
| 420 | NLB-NRD-NRD | 0.6 | 18.6 | 99.1 |
| 421 | C3N-O2A-S3N | 0.5 | 16.7 | 96.5 |
| 422 | C2P-NRD-O2A | 0.5 | 55.6 | 99.2 |
| 423 | C3P-CRN-NRD | 0.5 | 43.9 | 99.0 |
| 424 | C3N-C3N-S3N | 0.5 | 12.8 | 96.5 |
| 425 | C3N-NRD-O3B | 0.5 | 42.4 | 99.0 |
| 426 | C2P-C3N-C3P | 0.5 | 72.8 | 99.2 |
| 427 | C3N-NLB-NRD | 0.5 | 31.9 | 99.1 |
| 428 | C2P-CRN-O2A | 0.5 | 40.9 | 99.0 |
| 429 | C3N-C3N-OLC | 0.5 | 37.4 | 99.0 |
| 430 | NRD-NRD-NRE | 0.5 | 10.4 | 98.3 |
| 431 | C2P-C3N-CRN | 0.5 | 46.2 | 99.0 |
| 432 | C2P-C3N-S3N | 0.5 | 14.6 | 96.5 |
| 433 | C2P-C3N-NRE | 0.5 | 22.0 | 98.3 |
| 434 | C2P-NRD-NRE | 0.4 | 14.4 | 98.3 |
| 435 | C3N-O2A-OLC | 0.4 | 43.7 | 99.0 |
| 436 | C3N-NRD-OLC | 0.4 | 36.7 | 99.0 |
| 437 | C3N-C3P-NRD | 0.4 | 68.4 | 99.2 |
| 438 | CRN-NRD-O2A | 0.4 | 39.7 | 99.0 |
| 439 | C3N-C3N-C3P | 0.4 | 71.4 | 99.2 |
| 440 | C3N-NRD-S3N | 0.4 | 14.6 | 96.5 |
| 441 | C2P-C3N-OLC | 0.4 | 39.7 | 99.0 |
| 442 | C3N-C3N-O3B | 0.4 | 39.9 | 99.0 |
| 443 | C3N-NRD-O2A | 0.4 | 65.4 | 99.2 |
| 444 | C3N-C3N-C3N | 0.4 | 53.6 | 99.2 |
| 445 | C3N-C3N-O2A | 0.4 | 67.0 | 99.2 |
| 446 | C3N-NRD-NRE | 0.4 | 18.8 | 98.3 |
| 447 | C2P-C3N-O2A | 0.4 | 65.4 | 99.2 |
| 448 | C2P-C2P-C3N | 0.3 | 57.5 | 99.2 |
| 449 | C2P-CRN-NRD | 0.3 | 33.0 | 99.0 |
| 450 | C3N-NRD-NRD | 0.3 | 50.2 | 99.2 |
| 451 | C3N-CRN-NRD | 0.3 | 42.2 | 99.0 |
| 452 | CRN-NRD-NRD | 0.3 | 23.8 | 99.0 |
| 453 | C2P-C3N-C3N | 0.3 | 60.0 | 99.2 |
| 454 | C2P-C3N-NRD | 0.3 | 58.8 | 99.2 |
| 455 | C3N-C3N-NRD | 0.2 | 56.0 | 99.2 |

**Fig. S1.** **Parameter optimization results for the *Penalty* weight *W***. The success rates on the training set (a subset of LPDB, see Methods) when different *Penalty* weights were applied.

**Fig S2. Parameter optimization results for raising the lower thresholds.** The success rates on the training set (a subset of LPDB, see Methods) when the lower distance thresholds for calculating motif *Gains* were raised 0%, 10%, 20%, and 30%, higher than those used for calculating *Penalties*.

**Fig S3. Examples of some atom types in Table 1.**
